# Effects of ulcerative colitis and Crohn’s disease on neurodegenerative diseases: A Mendelian randomization study

**DOI:** 10.3389/fgene.2022.846005

**Published:** 2022-08-15

**Authors:** Hong Li, Zheng Wen

**Affiliations:** ^1^ Department of Medical Administration, Minzu Hospital of Guangxi Zhuang Autonomous Region, Nanning, China; ^2^ Department of Epidemiology, School of Public Health, Guangxi Medical University, Nanning, China

**Keywords:** ulcerative colitis, Crohn’s disease, Parkinson’s disease, Alzheimer’s disease, amyotrophic lateral sclerosis, Mendelian randomization, causal association

## Abstract

**Background:** Both ulcerative colitis (UC) and Crohn’s disease (CD) are associated with neurodegenerative diseases (NDs) in observational studies, but the causality remains controversial. We aimed to use Mendelian randomization (MR) analysis to explore causal associations between UC and CD and NDs.

**Methods:** We used single nucleotide polymorphisms (SNPs) associated (*p* < 5 × 10^−8^) with UC and CD as instrumental variables (IVs) to perform the MR analysis on the risks of three NDs, namely, Alzheimer’s Disease (AD), Parkinson’s Disease (PD), and Amyotrophic Lateral Sclerosis (ALS). The inverse variance weighted (IVW) was the primary method and supplement with the weighted median and MR-Egger regression. Moreover, the MR-Egger intercept test, Cochran’s Q test, and “leave one out” sensitivity analysis were implemented to assess the horizontal pleiotropy, heterogeneities, and stability of these SNPs on NDs. To verify the stability of the results, we re-run the MR analysis by using another set of IVs of UC and CD. A reverse causality analysis was conducted to test whether NDs were causally associated with UC or CD. The significance threshold was set at *p* < 0.05/6 = 0.008.

**Results:** In the primary MR analysis, the IVW method yielded no evidence to support a causal association between UC and PD (*OR*: 1.01, 95% *CI*: 0.96–1.06, *p* = 0.65), AD (*OR*: 1.00, 95% *CI*: 0.99–1.00, *p* = 0.57), or ALS (*OR*: 0.98, 95% *CI*: 0.96–1.01, *p* = 0.24), and neither did the MR-Egger and weighted median methods. Our MR analysis also suggested no definitively causal effect of the genetically predicted CD on PD (*OR*: 1.01, 95% *CI*: 0.97–1.05, *p* = 0.54), AD (*OR*: 1.00, 95% *CI*: 0.99–1.00, *p* = 0.26), or ALS (*OR*: 0.99, 95% *CI*: 0.96–1.02, *p* = 0.41), as well as MR-Egger and weighted median methods. Consistent results were found in validation analyses. We did not find a significant causal effect of NDs on UC or CD in the reverse MR analysis.

**Conclusion:** No evidence indicated an association between the risks of NDs and genetically predicted UC or CD. The MR results did not support a causal association between UC or CD and three NDs.

## Introduction

Neurodegenerative diseases (NDs) are a class of neurological disorders that involve progressive degeneration and/or loss of neurons from the central nervous system. They have become a considerable health burden in society because of the aging population. A significant number of NDs can be defined, but recent research focused on Alzheimer’s Disease [AD, the most common cause of dementia (Blennow et al., 2006)], Parkinson’s Disease (PD), Multiple System Atrophy, and Amyotrophic Lateral Sclerosis (ALS) (Przedborski et al., 2003). Unfortunately, although numerous research efforts on NDs are underway, the cause of the neurodegenerative process remains multifarious and complex, and there is currently no disease-specific cure.

In the past years, a large number of studies have linked intestinal disorders to NDs (Fu et al., 2020; Zhu et al., 2021). Inflammatory bowel disease (IBD) is a chronic recurrent intestinal inflammatory disorder, that includes ulcerative colitis (UC) and Crohn’s disease (CD) (Nakase et al., 2021). Although previous studies have explored the relationship between IBD (UC and CD) and the risk of some NDs, the association remains elusive and controversial. A population-based case-control study has shown an inverse association between PD with IBD (Camacho-Soto et al., 2018). However, three recent extensive cohort studies found that IBD was associated with an increased incidence of PD, suggesting an increased risk of PD in patients with underlying IBD (Lin et al., 2016; Villumsen et al., 2019b; Weimers et al., 2019). A previous study has shown that UC patients increased the risk of dying from AD (2.40, 95% *CI*: 1.00–5.76) (Caini et al., 2016). The population-based cohort study from Taiwan has reported a significant relationship between IBD (UC and CD) and subsequent development of dementia (AD, vascular dementia, and unspecified dementia) (Zhang B. et al., 2021). The previous research is limited to the relationship between IBD (UC and CD) and other NDs.

Results were controversial, and methodological limitations of observational studies, such as the potential for biases and confounding, remain a cause for concern. Thus, there is a demand to investigate the causal relationship by using a suitable method in large-scale samples. Mendelian randomization (MR) analysis (Davey Smith and Hemani, 2014; Richmond and Davey Smith, 2021) is a technique that uses genetic variants as instrumental variables (IVs) to evaluate the causal relationship between an exposure and an outcome. This technique can determine the causal effect and control for residual confounding when in the presence of conflicting observational evidence. We designed a two-sample MR analysis to examine whether UC or CD is causally associated with NDs (AD, PD, and ALS).

## Materials and methods

### Study design

**FIGURE 1:**
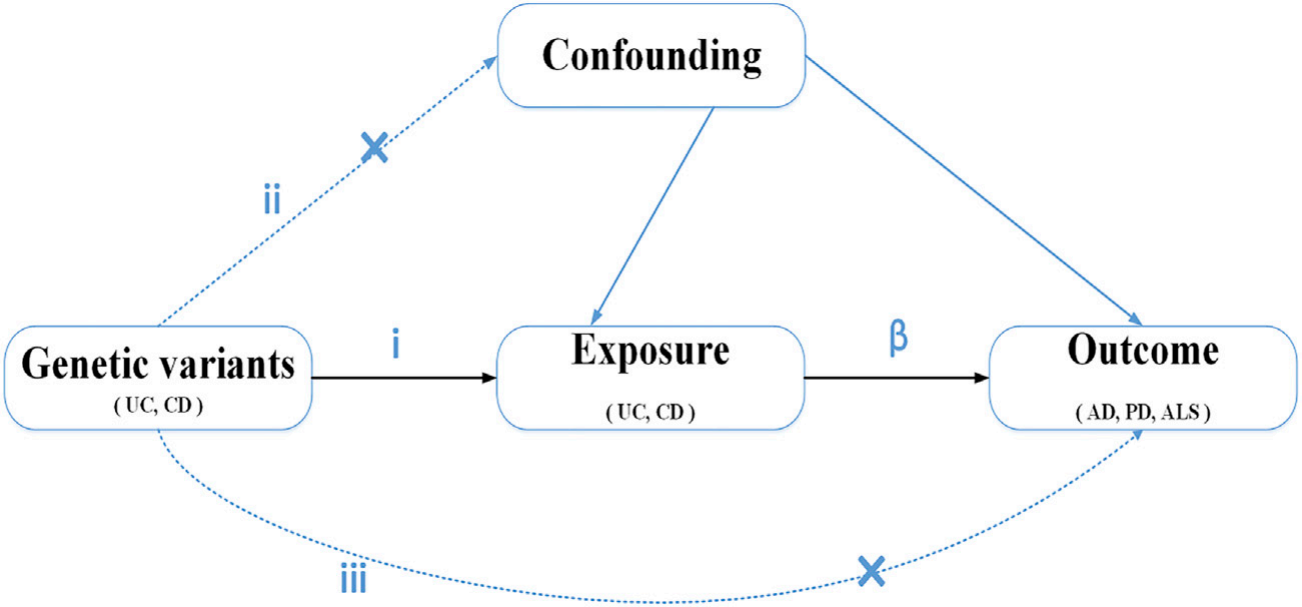
Diagram of Mendelian randomization study design: i) The genetic variants selected as instrumental variables (IVs) should be associated with the exposure (*P* < 5E-8). ii) genetic variants are not related to other confounder factors. iii) genetic variants would have an effect on the outcomes (ND) only *via* exposure (UC and CD), not *via* any alternative pathways. *β*, causal relationship.

We conducted a two-sample MR method to evaluate the causal effect of IBD (UC and CD) on NDs (Figure 1). MR is based on three key assumptions, as follows. (i) The genetic variants, which are chosen as IVs, are significantly associated with exposure (UC and CD). (ii) Genetic variants are unrelated to other confounder factors. (iii) Genetic variants affect the outcomes (NDs) only *via* exposure (UC and CD) and not *via* other pathways. If the three assumptions are true, the MR design provides a robust estimate of the causal effect and can control potential confounders and reverse causation (Lawlor et al., 2008). Data on the associations of SNPs with IBD and three NDs (AD, PD, and ALS) were based on the recently published and publicly available large-scale genome-wide association studies (GWAS).

### Data sources

Candidate genetic instruments for the exposure (UC and CD) were obtained from the GWAS summary data (Liu et al., 2015). The largest GWAS published to date for IBD was derived from an extended cohort of 96,486 individuals (including 86,640 European individuals and 9,846 non-Europeans), which was conducted by the International IBD Genetics Consortium. We just selected summary association statistics of UC (*n* = 27,432, cases/controls: 6,968/20,464) and CD (*n* = 20,883, cases/controls: 5,956/14,927) from the European GWAS (Liu et al., 2015), to prevent pleiotropic bias in cross-ancestral cases. In addition, we obtained previous summary data on UC and CD from GWAS conveyed by Jostins et al. (2012) (UC: 10,920 cases and 15,977 controls, CD: 14,763 cases and 15,977 controls) for validation purposes. All participants were of European ancestry. Full summary statistics for the exposure GWAS were downloaded from the IEU GWAS database (https://gwas.mrcieu.ac.uk/datasets/).

Outcome summary statistics of PD were extracted from one large-scale GWAS conducted by Nalls et al. The data for the meta-analysis were from 17 GWAS datasets (including the summary statistics published in Nalls et al., 2014, 23 and Me participants, and 13 new case-control sample series) (Nalls et al., 2019). We used a subset of the PD GWAS summary statistics (excluding 23 and Me participants), which is available from the International Parkinson’s Disease Genomics Consortium (https://pdgenetics.org/resources), including data from 33,674 PD cases and 449,056 controls (Nalls et al., 2019). GWAS data on AD were accessed from the recently published genome-wide meta-analysis (https://ctg.cncr.nl/software/summary_statistics) and included 71,880 AD cases and 383,378 controls (Jansen et al., 2019). For ALS, the current study was based on European ancestry populations, and was the largest genome-based GWAS to date; it can be extracted from the ALS Variant Server (http://als.umassmed.edu). It include 20,806 ALS cases and 59,804 controls (Nicolas et al., 2018). We also could download GWAS summary statistics of PD and ALS from the publicly available IEU GWAS database (https://gwas.mrcieu.ac.uk/datasets/).

### Selection of genetic variants

To ensure a strong relationship between IVs and UC and CD, we extracted SNPs that are significantly associated with UC and CD (*p* < 5 × 10^−8^) from the corresponding GWAS summary data (Jostins et al., 2012; Liu et al., 2015). It is important to ensure that all the selected IVs for UC and CD are not in linkage disequilibrium (LD), because using SNPs in strong LD may cause biased results in a Mendelian randomization analysis. We used the PLINK clumping method (*R* ^*2*^ < 0.001, window size = 10,000 kb) based on European samples to calculate the LD through the two-sample MR package. When LD *R* ^*2*^ > 0.001, only the SNP with the lower *p*-value was retained. Subsequently, we extracted data for the remaining SNPs from the outcome trait (NDs) GWAS summary. The SNP that is directly associated with NDs (*p* < 5 × 10^−8^) was precluded. If an IV not available from the target dataset, it was excluded. Finally, to assure that the effect alleles belong to the same allele, we excluded ambiguous SNPs with incompatible alleles (e.g., A/G vs. A/C) and palindromic SNPs with ambiguous alleles (e.g., A/T vs. G/C) through harmonized the exposure and outcome datasets (Hartwig et al., 2016).

According to MR analysis demand, the selected IVs need to have a strong relationship with exposure (UC and CD). The strength of the IVs was estimated on the basis of the *F* statistic. Finally, we used the formula $F=\frac{R^{2}\left(n\;-\;k\;-\;1\right)}{k\left(\;1\;-\;R^{2}\right)}$ (*R* ^*2*^: variance of exposure explained by selected instrumental variables, *n*: sample size, *k*: number of instrumental variables) to calculate the *F* statistic. *R* ^*2*^ was calculated by the following formula: $R^{2}=\sum_{i\;=\;1}^{K}\frac{\beta_{i}^{2}}{\beta_{i}^{2}+\;2\;*\;n\;*\;se\;{\left(\beta_{i}\right)}^{2}}$ [*β*_*i*_: effect size for *SNP*_*i*_, *se*(*β*_*i*_): standard error for *SNP*_*i*_, *n:* sample size for *SNP*_*i*_, *K*: number of the selected genetic variants] (Shim et al., 2015). A higher *F* statistic corresponded to a smaller bias (Burgess and Thompson, 2011). If *F* > 10, then the weak instrumental variable bias had a slim chance (Staiger and Stock, 1997).

### Mendelian randomization analysis

MR analysis is well established and widely used to estimate the causative effect of exposure variables on an outcome by using genetic instruments as IVs. We performed MR analysis using the above-described SNP genetic instruments. Effect sizes and standard errors were obtained for each SNP from NDs GWAS summary statistics. We applied the inverse variance weighted (IVW) as the primary method and supplemented it with the weighted median (Bowden et al., 2016a) and MR-Egger regression (Bowden et al., 2016b) approaches to perform the MR analysis. These methods were grounded on various assumptions and helped examine each other’s robustness. IVW method was based on an inverse-variance weighted formula and was used to estimate the combined causal effects. At the same time, it was used to minimize the variance of the weighted average (Lee et al., 2016). IVW analysis assumed that each genetic variant was a valid instrumental variable. The MR-Egger regression method was robust to invalid instruments, which estimated the effects by adjusting for horizontal pleiotropy when instrumental variables affected NDs *via* other biological pathways. The weighted median regression method calculated the robust causal effects. This method, is based on the assumption that up to 50% of the selected SNPs were valid instrumental variables.

The MR-Egger method provides a reliable estimation for the IVs assumption and can be used to check for the presence of potential pleiotropy (Burgess and Thompson, 2017). Therefore, the MR-Egger was used to assess potential pleiotropic relationships between the IVs and confounders. We performed a heterogeneity test by using Cochran’s Q test. If obvious heterogeneities existed, MR pleiotropy residual sum and outlier (MR-PRESSO) method was used to confirm the horizontal pleiotropic outliers. Then, the outliers were removed, and MR analysis repetition was conducted. On the side, a sensitivity analysis was conducted to evaluate the robustness of the causal estimates. A “leave one out” analysis was performed to examine the possibility that causal association was being driven by a single SNP.

**FIGURE 2:**
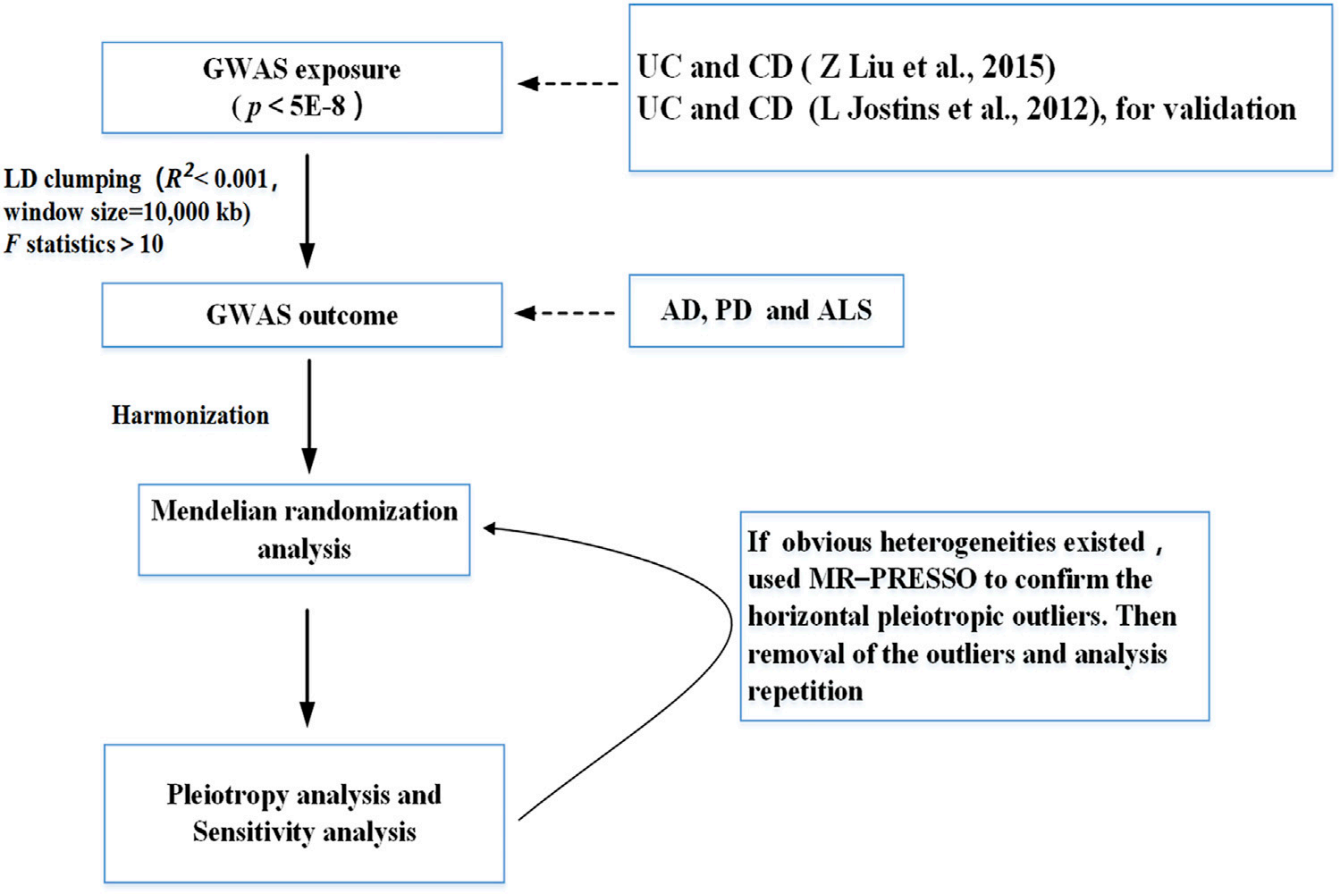
Flow chart of this Mendelian randomization study. UC, ulcerative colitis; CD, Crohn’s disease; AD, Alzheimer’s Disease; PD, Parkinson’s Disease; ALS, Amyotrophic Lateral Sclerosis.

The statistical power in MR analysis for each association was calculated *via* the tool for binary outcomes (http://cnsgenomics.com/shiny/mRnd/) (Brion et al., 2013). We used the two-sample MR and MR-PRESSO packages for the MR analyses. The significance threshold was set at *p* < 0.05/X/Y = 0.05/2/3 = 0.008, corrected by the Bonferroni method (X: the number of exposures, Y: the number of outcomes). R4.1.1 (R Project for Statistical Computing) was used for all analyses. Figure 2 presents the flowchart that illustrates our study’s process.

### Reverse causality

We extracted SNPs that are significantly associated with NDs (*p* < 5 × 10^−8^) from the corresponding GWAS summary data (Nicolas et al., 2018; Jansen et al., 2019; Nalls et al., 2019). Next, selecting independent SNPs (*R* ^*2*^ < 0.001, window size = 10,000 kb) and strong IVs (*F* statistics > 10) to study reverse causality to test whether NDs were causally associated with UC or CD. We performed MR analyses following the above-described methods (IVW, MR-Egger regression and weighted median regression) to study the causal relationship.

## Results

### Selection of instrumental variables

SNPs that strongly (*p* < 5 × 10^−8^) associated with UC and CD were identified from the corresponding GWAS summary data. To examine assumptions *i* and *ii*, we tested whether any of the picked SNPs were affected by LD. We selected the SNP with the lowest *p*-value for association with UC and CD if genetic variants were in LD. In addition, we excluded the SNP that was palindromic or directly associated with NDs (PD, AD, and ALS) (*p* < 5 × 10^−8^). A full description of the significant variants (after LD pruning) for exposure (UC and CD) is shown in Supplementary Tables S1–S4. The *F* statistic value was from 23.8 to 59.5, thereby demonstrating a low risk of week instrument bias (Supplementary Table S8). Our MR analysis yielded sufficient power at an alpha rate of 5% (above 85% to detect an *OR* of 1.10) to find moderate relationships between UC/CD and NDs. For reverse causality, we incorporated 23, 27, and 6 independent SNPs with significant *p*-value less than 5 × 10^−8^ as IV SNPs for PD, AD and ALS (Supplementary Tables S5–S7).

### Associations of ulcerative colitis on neurodegenerative diseases risk

**FIGURE 3:**
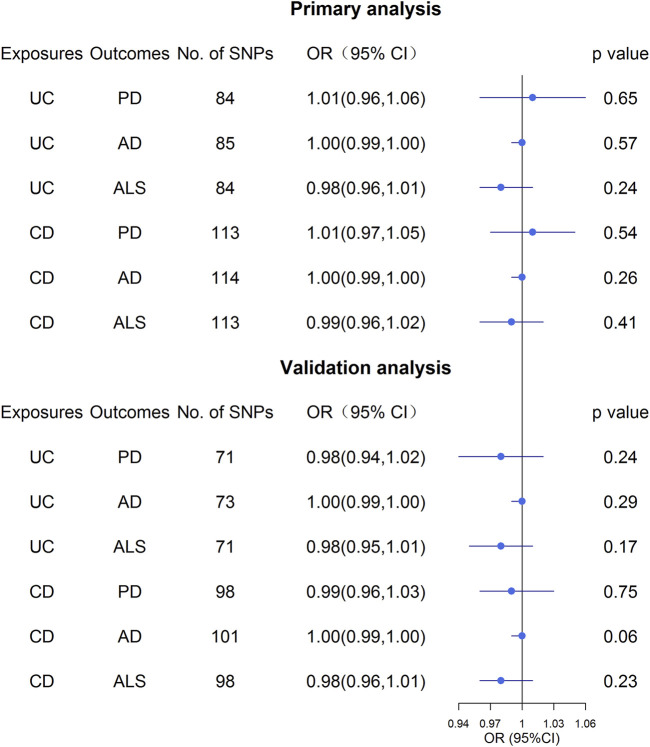
Effect estimates of UC and CD on NDs in the Mendelian randomization study. UC, ulcerative colitis; CD, Crohn’s disease; AD, Alzheimer’s Disease; PD, Parkinson’s Disease; ALS, Amyotrophic Lateral Sclerosis.

**FIGURE 4:**
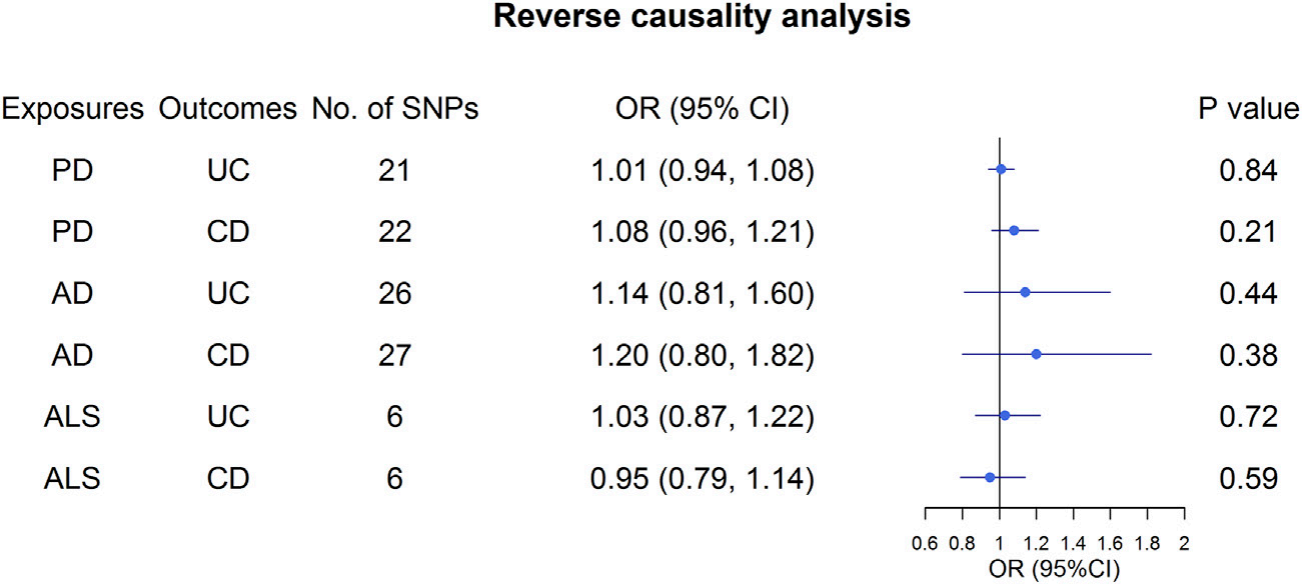
Effect estimates of NDs on UC and CD in the Mendelian randomization study. UC, ulcerative colitis; CD, Crohn’s disease; AD, Alzheimer’s Disease; PD, Parkinson’s Disease; ALS, Amyotrophic Lateral Sclerosis.

Overall, the MR results did not support the relationship between UC and the three NDs (Figure 3; Supplementary Table S9). In the primary analysis, using the IVW method, we found that UC was not causally related to PD (*OR*: 1.01, 95% *CI*: 0.96–1.06, *p* = 0.65), AD (*OR*: 1.00, 95% *CI*: 0.99–1.00, *p* = 0.57), or ALS (*OR*: 0.98, 95% *CI*: 0.96–1.01, *p* = 0.24) (Figure 3; Supplementary Table S9). Similarly, the MR-Egger regression and the weighted median approach yielded no evidence to support a causal association between UC and NDs (Supplementary Table S9). Furthermore, the intercept term from MR-Egger regression was null for UC and NDs (all *p* for intercept >0.05; Supplementary Table S9), which demonstrated that directional pleiotropy did not excessively influence the results. Cochran’s Q test revealed evidence of heterogeneity among the effects of UC-associated SNPs on the NDs (Supplementary Table S9). Moreover, statistically significant heterogeneity existed in the effect of UC-associated SNPs on PD (IVW: *Q* = 174.96, *p* = 1.59 × 10^−8^, MR-Egger: *Q* = 174.59, *p =* 1.19 × 10^−8^. Next, we conducted a repetitionary MR analysis after removing the outliers (rs1801274: chr1:161479745, rs76904798: chr12:40614434, and rs1297256: chr21:16805676) identified by MR-PRESSO. Despite the null casual relationship between UC and PD remaining after excluding outliers, evidence of heterogeneity existed through Cochran’s Q test (Supplementary Table S9). In the sensitivity analyses, results from the “leave one out” analysis provided support that no single SNP was driving the IVW point estimate (Supplementary Figures S1–S3). Moreover, in the validation analysis, the MR results also revealed that UC was not causally related to NDs (Figure 3; Supplementary Table S10). As for reverse causality, there were no causal associations between genetically predicted NDs and UC risk (Figure 4; Supplementary Table S11).

### Associations of Crohn’s disease on neurodegenerative diseases risk

Similar to the analysis at UC on NDs, we found no causal relationship between CD and PD (*OR*: 1.01, 95% *CI*: 0.97–1.05, *p* = 0.54), AD (*OR*: 1.00, 95% *CI*: 0.99–1.00, *p* = 0.26) or ALS (*OR*: 0.99, 95% *CI*: 0.96–1.02, *p* = 0.41) in IVW analysis (Figure 3; Supplementary Table S9). This finding was also stable in other MR analysis methods, including weighted median and the MR-Egger analyses. In addition, no directional pleiotropy effects were discovered according to the MR-Egger intercept (all *p* for intercept >0.05; Supplementary Table S9). There was significant evidence of heterogeneity, and MR-PRESSO identified five outliers, namely, rs11175963 (chr12:40702771), rs7194886 (chr16:50725193), rs780094 (chr2:27741237), rs1297258 (chr21:16806709), and rs727563 (chr22:41867377), in the analysis of CD on PD. CD was still not associated with the PD after excluding the outliers (Supplementary Table S9). The results of the “leave one out” analysis indicated that no causal effect exists (Supplementary Figures S4–S6). In the same way, the MR results in the validation analysis were consistent with the primary analysis, indicating that CD was not causally related to NDs (Supplementary Table S10). In the reverse MR analysis, the IVW, weighted median, and the MR-Egger analyses results showed that genetically higher risks of NDs had no causal effect on UC or CD risk (Figure 4; Supplementary Table S11).

## Discussion

We utilized the MR design to investigate the causal relationship between genetically predicted UC and CD and the risk of three common NDs, namely, AD, PD, and ALS. The results from three different estimation methods of MR analyses suggested that UC or CD did not play a role in the development of AD, PD, or ALS. The reverse MR analysis similarly found no evidence that genetic liability to NDs was related to UC or CD.

UC and CD are two forms of IBD, which is a chronic state of dysregulated inflammation that onsets at a young age (Kaser et al., 2010). In the past decade, several population-based observational studies have tried to examine the association of IBD and AD and PD, whereas the association between IBD and ALS has rarely been explored. Two retrospective longitudinal studies that included participants from the Taiwan National Health Insurance (NHI) programme demonstrated that the incidence of PD and AD were linked to UC and CD (Lin et al., 2016; Zhang B. et al., 2021). In addition, the latest population-based study reported that IBD was independently associated with the development of AD, and the association was stronger in patients with CD (adjusted *OR* = 3.34, 95% *CI*: 3.25–3.42) (Aggarwal et al., 2022). Moreover, one meta-analysis declared that the *OR* for IBD with an increased risk of PD was 1.16 (95% *CI*, 0.89–1.52), and the *OR* for IBD with AD was 2.40 (95% *CI*, 1.00–5.76) (Fu et al., 2020). And the latest meta-analysis revealed that the risk of AD was higher in IBD patients (*RR* = 2.79, 95% *CI*: 1.1–7.04; *p* < 0.001) (Zhang et al., 2022). Another meta-analysis of nine observational studies (6 cohort studies, three case-control studies and one cross-sectional study) with a total of 12,177,520 patients showed that ulcerative colitis (adjusted *HR* = 1.25, 95% *CI*: 1.13–1.38) and Crohn’s disease (adjusted *HR* = 1.33, 95% *CI*: 1.21–1.45) could increase the risk of PD (Zhu et al., 2022). An earlier systematic review and meta-analysis also showed a positive association that CD had a 28% increased risk of PD, and UC had a 30% increased risk of PD compared with the controls (Zhu et al., 2019).

However, the results of observational studies are conflicting. Several high-quality studies with large samples have also found null or negative associations between IBD and NDs (Camacho-Soto et al., 2018; Coates et al., 2021; Kim et al., 2021). A longitudinal cohort study of U.S. older adults reported that immune-mediated inflammatory diseases (including rheumatoid arthritis, psoriatic arthritis, ankylosing spondylitis, CD, UC, and related conditions) do not have an increased risk of AD over a 6-year period (Booth et al., 2021). And a new meta-analysis demonstrated an aHR of 1.47 for dementia in UC patients, but the statistics were not significant (95% *CI*: 0.95–2.82, *p* = 0.81) (Zuin et al., 2022). A case-control conducted by Camacho-Soto et al. (2018) found an inverse association between PD and CD (*OR* = 0.83, 95% CI 0.74–0.93) and UC (OR = 0.88, 95% CI: 0.82–0.96). In addition, a nationwide population-based cohort that included 24,830 IBD patients and 99,320 non-IBD controls published recently found that the risk of PD was not significantly higher in CD patients (adjusted *HR*, 1.03; 95% *CI*, 0.58–1.84) (Kim et al., 2021). It is similar to the cohort from Denmark that the increased risk of parkinsonism was not significantly different among patients with CD (HR = 1.12; 95% *CI* 0.89–1.40) (Villumsen et al., 2019b).

Owing to the difference of methods used to diagnose NDs, the individual observational population, or the definition of IBD (UC and CD), and there was statistical heterogeneity for the meta-analysis to evaluate the association between IBD (UC and CD) and NDs. The statistical heterogeneity can make the interpretation of meta-analyzed findings difficult. The causality remains unclear, because it may be a result of bias or various confounders inherent to observational studies. For instance, surveillance bias could have played a role in the positive association between IBD and PD (Villumsen et al., 2019a). And the gut microbiota may be a common risk for both IBD and NDs, which may provide a possible confounder in the positive relationship for both diseases. Gut microbiota or metabolites was found to be a risk factor for both IBD (Zhang Z. et al., 2021) and NDs (Ning et al., 2022) in previous MR studies. Moreover, the shared genetic loci between IBD and NDs may be linked to the increased susceptibility to both diseases. For example, leucine-rich repeat kinase 2 (LRRK2) was initially identified as a causal gene in PD and has recently been associated with an increased incidence of CD (Herrick and Tansey, 2021).

One major strength of our study is its two-sample MR design, which could circumvent the limitations of observational studies with measurement errors and residual confounding. It can minimize reverse causation bias, thereby affording high-quality evidence. Nevertheless, our study has several limitations. One limitation is that residual pleiotropy might remain, despite the range of sensitivity analyses conducted to explore and account for pleiotropy. However, no evidence of horizontal pleiotropy was found based on the intercept estimates of the MR-Egger analysis. Moreover, we failed to stratify the causal effects between UC or CD and NDs by gender or age. Meanwhile, the database used in the MR analysis originated from European-ancestry studies; thus, we needed to be prudent when generalizing the finding to other populations. The results will provide a deeper understanding of whether future studies are carried out on other non-European populations.

## Conclusion

In this study, we found no evidence indicating an association between NDs (AD, PD, and ALS) risk and genetically predicted UC or CD. The previous associations between UC or CD and NDs (AD, PD, and ALS) may result from potential biases.

## Data Availability

The original contributions presented in the study are included in the article/Supplementary Material, further inquiries can be directed to the corresponding author.
