# Supplementary material for: Effects of ulcerative colitis and Crohn’s disease on neurodegenerative diseases: A Mendelian randomization study
Source: Front Genet. 2022 Aug 15;13:846005. doi: 10.3389/fgene.2022.846005 (PMC9421062; doi:10.3389/fgene.2022.846005)
Supplement: Supplementary file 1 [file DataSheet1.PDF]

Supplementary Material

Table S1. Characteristics of selected SNPs for ulcerative colitis.
Table S2. Characteristics of selected SNPs for Crohn's disease.
Table S3. Characteristics of selected SNPs for ulcerative colitis (for validation).
Table S4. Characteristics of selected SNPs for Crohn's disease (for validation).
Table S5. Characteristics of selected SNPs for Parkinson's Disease.
Table S6. Characteristics of selected SNPs for Alzheimer's Disease.
Table S7. Characteristics of selected SNPs for Amyotrophic Lateral Sclerosis.
Table S8. *F*-statistics for the exposure variables.
Table S9. Associations between genetically predicted inflammatory bowel disease (ulcerative colitis and Crohn's disease) and neurodegenerative diseases in Mendelian randomization analyses.
Table S10. Associations between genetically predicted inflammatory bowel disease (ulcerative colitis and Crohn's disease, for validation) and neurodegenerative diseases in Mendelian randomization analyses.
Table S11. Associations between genetically predicted neurodegenerative diseases and ulcerative colitis and Crohn's disease in Mendelian randomization analyses.
Figure S1. Leave-one-out plot presenting the relationship between ulcerative colitis and Parkinson's Disease.
Figure S2. Leave-one-out plot presenting the relationship between ulcerative colitis and Alzheimer's Disease.
Figure S3. Leave-one-out plot presenting the relationship between ulcerative colitis and Amyotrophic Lateral Sclerosis.
Figure S4. Leave-one-out plot presenting the relationship between Crohn's disease and Parkinson's Disease.
Figure S5. Leave-one-out plot presenting the relationship between Crohn's disease and Alzheimer's Disease.
Figure S6. Leave-one-out plot presenting the relationship between Crohn's disease and Amyotrophic Lateral Sclerosis.

Table S1. Characteristics of selected SNPs for ulcerative colitis.

SNP	chr: pos	effect_allele	other_allele	beta	se	eaf	samplesize	*P*-value
rs2274351	chr10:104264107	T	C	0.07	0.01	0.54	27432	4.90E-08
rs3776414	chr5:10689562	G	T	0.07	0.01	0.38	27432	4.10E-08
rs13255292	chr8:129076573	T	C	-0.08	0.01	0.33	27432	3.82E-08
rs11083840	chr19:47119910	G	T	0.07	0.01	0.40	27432	3.41E-08
rs4973341	chr2:228660362	T	C	0.07	0.01	0.66	27432	2.25E-08
rs4712520	chr6:20640871	C	T	0.09	0.02	0.82	27432	2.21E-08
rs16841904	chr1:197701992	T	C	0.09	0.02	0.20	27432	1.90E-08
rs11230563	chr11:60776209	T	C	-0.08	0.01	0.35	27432	1.90E-08
rs10460566	chr2:25483121	A	G	-0.08	0.01	0.76	27432	1.60E-08
rs7404095	chr16:23864590	C	T	0.07	0.01	0.58	27432	1.52E-08
rs59418206	chr10:35331624	A	G	0.07	0.01	0.35	27432	1.45E-08
rs79045992	chr16:68518992	A	G	0.12	0.02	0.10	27432	1.43E-08
rs12718244	chr7:50175654	A	G	0.07	0.01	0.41	27432	1.41E-08
rs13430791	chr2:43481013	A	G	0.11	0.02	0.12	27432	1.39E-08
rs913678	chr20:48955424	C	T	-0.08	0.01	0.33	27432	1.23E-08
rs11229555	chr11:58408687	T	G	-0.08	0.01	0.25	27432	1.21E-08
rs2497318	chr10:94432000	T	C	-0.07	0.01	0.45	27432	1.15E-08
rs36070529	chr5:158619835	A	G	-0.09	0.02	0.20	27432	1.04E-08
rs4747886	chr10:6176166	T	C	0.07	0.01	0.41	27432	9.58E-09
rs1077773	chr7:17442679	A	G	0.07	0.01	0.52	27432	5.96E-09
rs4743820	chr9:93928416	T	C	0.08	0.01	0.70	27432	4.05E-09
rs4656958	chr1:160856964	G	A	0.08	0.01	0.68	27432	2.82E-09
rs76904798	chr12:40614434	T	C	0.10	0.02	0.14	27432	2.78E-09
rs4976646	chr5:176788570	C	T	0.08	0.01	0.34	27432	2.52E-09
rs11676348	chr2:219010146	T	C	0.07	0.01	0.48	27432	2.08E-09
rs55808324	chr14:88444752	A	G	0.13	0.02	0.09	27432	1.47E-09
rs12103	chr1:1247494	C	T	-0.10	0.02	0.82	27432	9.96E-10
rs11641184	chr16:11704651	A	C	0.08	0.01	0.48	27432	4.24E-10
rs4947328	chr6:31561747	G	A	0.24	0.04	0.02	27432	3.38E-10
rs11150589	chr16:30482494	C	T	-0.08	0.01	0.53	27432	3.28E-10
rs483905	chr11:96023427	A	G	0.08	0.01	0.29	27432	3.16E-10
rs7240004	chr18:46395022	G	A	-0.08	0.01	0.38	27432	2.50E-10
rs13136827	chr4:123171318	C	T	-0.11	0.02	0.16	27432	2.35E-10
rs1990760	chr2:163124051	T	C	-0.09	0.01	0.61	27432	1.78E-10
rs76546301	chr7:50498389	A	G	0.26	0.04	0.02	27432	1.05E-10
rs111830527	chr1:22687173	A	G	-0.19	0.03	0.05	27432	5.09E-11
rs9891119	chr17:40507980	C	A	-0.09	0.01	0.35	27432	1.72E-11
rs12720356	chr19:10469975	C	A	0.15	0.02	0.09	27432	1.67E-11
rs10910092	chr1:2501516	G	A	-0.09	0.01	0.47	27432	1.42E-11
rs17694108	chr19:33731551	A	G	0.10	0.01	0.28	27432	6.17E-12
rs7711427*	chr5:40414886	C	A	0.09	0.01	0.61	27432	3.67E-12
rs17780256	chr17:70642923	C	A	-0.12	0.02	0.19	27432	6.13E-13
rs2516440	chr6:31440497	A	G	-0.10	0.01	0.32	27432	4.40E-13
rs8096327	chr18:12887750	G	A	0.09	0.01	0.38	27432	2.24E-13
rs941823	chr13:41013977	C	T	0.11	0.01	0.75	27432	1.39E-13

rs3774937	chr4:103434253	C	T	0.10	0.01	0.33	27432	4.61E-14
rs9941524	chr2:199499443	G	A	0.10	0.01	0.46	27432	2.15E-14
rs4728142	chr7:128573967	A	G	0.10	0.01	0.44	27432	1.92E-14
rs10185424	chr2:102662888	G	T	-0.10	0.01	0.54	27432	1.47E-14
rs1182188	chr7:2869985	C	T	-0.11	0.01	0.30	27432	5.03E-15
rs9611131	chr22:39662480	C	T	-0.14	0.02	0.15	27432	3.84E-15
rs1297256	chr21:16805676	T	C	-0.10	0.01	0.42	27432	2.10E-15
rs35223180	chr1:8185902	T	G	-0.14	0.02	0.18	27432	1.04E-15
rs4456788	chr21:45616324	A	G	-0.10	0.01	0.61	27432	7.07E-16
rs4812833	chr20:43068996	A	G	0.10	0.01	0.52	27432	1.87E-16
rs1359946	chr13:27536972	A	G	0.13	0.02	0.18	27432	9.64E-17
rs34659678	chr6:111888540	T	C	0.21	0.03	0.06	27432	5.95E-17
rs6062496	chr20:62329099	A	G	0.11	0.01	0.57	27432	9.14E-19
rs4366152	chr9:117564875	C	T	0.12	0.01	0.68	27432	7.79E-19
rs4676410	chr2:241563739	A	G	0.14	0.02	0.20	27432	1.85E-19
rs661054	chr11:114430410	G	A	-0.12	0.01	0.34	27432	3.18E-20
rs10761659	chr10:64445564	G	A	0.12	0.01	0.54	27432	1.50E-20
rs9271858	chr6:32595223	G	A	0.12	0.01	0.51	27432	2.42E-21
rs6920220	chr6:138006504	A	G	0.15	0.02	0.21	27432	4.78E-22
rs61893460	chr11:76291154	A	G	0.12	0.01	0.44	27432	4.60E-22
rs7608910	chr2:61204856	G	A	0.13	0.01	0.39	27432	1.25E-23
rs4380874	chr7:107480315	C	T	-0.13	0.01	0.59	27432	6.43E-25
rs272882	chr5:131669161	T	G	0.15	0.01	0.67	27432	6.67E-26
rs11793497	chr9:139271850	G	A	0.13	0.01	0.42	27432	1.74E-26
rs56167332	chr5:158827769	A	C	0.14	0.01	0.34	27432	7.27E-27
rs7738430	chr6:31508836	C	T	0.37	0.03	0.03	27432	3.51E-27
rs10758669	chr9:4981602	A	C	-0.14	0.01	0.65	27432	1.04E-28
rs4795397	chr17:38023745	G	A	0.14	0.01	0.47	27432	1.01E-28
rs59655222	chr1:200875897	C	T	-0.17	0.01	0.28	27432	3.81E-31
rs12796489#	chr11:3059360	A	C	-0.68	0.06	0.02	27432	1.22E-33
rs12318183	chr12:68503836	A	C	0.16	0.01	0.39	27432	1.44E-37
rs9836291	chr3:49697459	A	G	0.17	0.01	0.29	27432	8.20E-38
rs10748783	chr10:101285872	A	C	-0.16	0.01	0.52	27432	7.73E-39
rs1801274	chr1:161479745	G	A	-0.17	0.01	0.50	27432	1.43E-41
rs6111031	chr20:1682037	T	C	-0.26	0.02	0.16	27432	1.33E-42
rs3024493	chr1:206943968	A	C	0.23	0.02	0.16	27432	1.42E-43
rs2836883	chr21:40466744	A	G	-0.23	0.01	0.27	27432	1.47E-53
rs7547569	chr1:67731368	C	T	-0.50	0.03	0.07	27432	8.71E-65
rs6426833	chr1:20171860	A	G	0.23	0.01	0.54	27432	3.77E-76
rs9271255	chr6:32580357	T	C	-0.28	0.01	0.73	27432	1.31E-94

Note: *For rs7711427, not available from the outcome summary statistics of Parkinson's Disease; #for rs12796489, not available from the outcome summary statistics of Amyotrophic Lateral Sclerosis; chr:pos, position according to GRCh37/hg19 genome assembly.

Abbreviations: SNP, single nucleotide polymorphism; se, standard error; eaf, effect allele frequency.

Table S2. Characteristics of selected SNPs for Crohn's disease.

SNP	chr: pos	effect_allele	other_allele	beta	se	eaf	samplesize	*P*-value
rs4703855	chr5:71693899	T	C	-0.07	0.01	0.30	20883	3.03E-08
rs915286	chr13:40695992	A	G	0.07	0.01	0.55	20883	2.59E-08
rs3184504	chr12:111884608	C	T	-0.07	0.01	0.51	20883	1.71E-08
rs1267501	chr6:14715257	C	T	0.09	0.02	0.81	20883	9.69E-09
rs7236492	chr18:77220616	T	C	-0.10	0.02	0.15	20883	9.09E-09
rs10798069	chr1:186875459	T	G	-0.07	0.01	0.49	20883	4.25E-09
rs17694108	chr19:33731551	A	G	0.08	0.01	0.28	20883	3.29E-09
rs17391694	chr1:78623626	T	C	-0.12	0.02	0.12	20883	2.62E-09
rs259964	chr20:57824309	G	A	-0.07	0.01	0.54	20883	2.08E-09
rs9594766	chr13:43040043	A	G	-0.07	0.01	0.53	20883	1.39E-09
rs640466	chr19:34670725	C	T	-0.08	0.01	0.37	20883	1.31E-09
rs7773324	chr6:382559	A	G	0.08	0.01	0.60	20883	1.06E-09
rs2538470	chr7:148220448	G	A	-0.07	0.01	0.64	20883	1.05E-09
rs7969592	chr12:68579649	G	A	-0.07	0.01	0.47	20883	1.04E-09
rs35320439	chr2:242737341	C	T	0.08	0.01	0.31	20883	9.89E-10
rs7786444	chr7:28154384	T	C	0.11	0.02	0.12	20883	9.83E-10
rs2641348	chr1:120437884	G	A	-0.12	0.02	0.11	20883	9.65E-10
rs7015630	chr8:90875918	C	T	-0.08	0.01	0.27	20883	9.00E-10
rs303429	chr10:30708441	T	C	0.08	0.01	0.60	20883	8.38E-10
rs71624119	chr5:55440730	A	G	-0.09	0.01	0.24	20883	6.57E-10
rs2974935	chr1:155181843	T	G	0.08	0.01	0.49	20883	5.80E-10
rs559928	chr11:64150370	C	T	0.10	0.02	0.81	20883	3.75E-10
rs2790241	chr10:60015313	T	G	-0.10	0.02	0.22	20883	3.29E-10
rs6827756	chr4:123184411	C	T	-0.08	0.01	0.63	20883	3.27E-10
rs2395022	chr7:98750379	C	A	-0.18	0.03	0.96	20883	3.13E-10
rs17129991	chr1:67862986	T	C	-0.28	0.05	0.02	20883	2.81E-10
rs727563	chr22:41867377	T	C	-0.09	0.01	0.80	20883	1.88E-10
rs36016881	chr1:8051241	G	A	-0.11	0.02	0.18	20883	1.60E-10
rs1517352	chr2:191931464	C	A	0.08	0.01	0.60	20883	1.31E-10
rs1847472	chr6:90973159	A	C	-0.09	0.01	0.34	20883	1.09E-10
rs6738394	chr2:219110625	A	G	0.08	0.01	0.45	20883	8.98E-11
rs9554587	chr13:100040654	G	A	-0.10	0.01	0.22	20883	8.29E-11
rs1569328	chr14:75741751	T	C	-0.11	0.02	0.17	20883	6.47E-11
rs11175963	chr12:40702771	T	C	-0.16	0.02	0.07	20883	4.83E-11
rs7438704	chr4:48363245	G	A	0.08	0.01	0.64	20883	3.42E-11
rs3129871	chr6:32406342	C	A	-0.09	0.01	0.66	20883	1.80E-11
rs11691685	chr2:145481827	G	A	-0.16	0.02	0.08	20883	1.35E-11
rs28999107	chr12:6493100	T	G	0.09	0.01	0.44	20883	1.29E-11
rs56163845	chr5:173373948	G	A	-0.09	0.01	0.31	20883	9.40E-12
rs72727394	chr15:38847022	T	C	0.10	0.01	0.20	20883	5.28E-12
rs6908425	chr6:20728731	C	T	0.10	0.02	0.78	20883	4.81E-12
rs6074022	chr20:44740196	T	C	-0.10	0.01	0.75	20883	2.70E-12
rs6740462	chr2:65667272	A	C	0.10	0.01	0.74	20883	1.74E-12
rs10800309	chr1:161472158	G	A	-0.09	0.01	0.66	20883	8.48E-13
rs3776414	chr5:10689562	G	T	0.09	0.01	0.38	20883	5.04E-13

rs2227551	chr10:75669190	T	G	0.10	0.01	0.73	20883	4.72E-13
rs61839660	chr10:6094697	T	C	0.15	0.02	0.09	20883	3.19E-13
rs79980175	chr5:40521892	C	A	-0.13	0.02	0.14	20883	1.70E-13
rs34804116	chr5:72539850	A	C	-0.09	0.01	0.39	20883	1.27E-13
rs1456896	chr7:50304461	T	C	0.10	0.01	0.69	20883	1.03E-13
rs11159833	chr14:88476004	T	C	0.16	0.02	0.09	20883	7.59E-14
rs3801810	chr7:26892531	A	G	0.11	0.01	0.23	20883	6.63E-14
rs1292053	chr17:57963537	G	A	0.09	0.01	0.44	20883	1.75E-14
rs11713774	chr3:18765978	C	T	0.13	0.02	0.14	20883	1.09E-14
rs2488389	chr1:197631141	A	G	0.11	0.01	0.21	20883	8.59E-15
rs181826	chr5:141526057	A	C	0.10	0.01	0.63	20883	4.53E-15
rs1363907	chr5:96252803	A	G	0.10	0.01	0.42	20883	3.89E-16
rs6651252	chr8:129567181	C	T	-0.15	0.02	0.13	20883	3.86E-16
rs34787213	chr11:60799046	T	C	-0.15	0.02	0.14	20883	2.85E-16
rs77981966	chr2:43777964	T	C	0.18	0.02	0.07	20883	2.19E-16
rs212388	chr6:159490436	T	C	-0.10	0.01	0.60	20883	1.80E-16
rs6456426	chr6:21438889	A	C	-0.10	0.01	0.50	20883	1.37E-16
rs1646019	chr16:11359680	T	C	-0.11	0.01	0.30	20883	8.62E-17
rs2284553	chr21:34776695	G	A	0.10	0.01	0.59	20883	5.63E-17
rs6679677	chr1:114303808	A	C	-0.19	0.02	0.10	20883	4.67E-17
rs9491892	chr6:128280358	G	T	0.14	0.02	0.15	20883	3.80E-17
rs12949918	chr17:40526273	C	T	-0.10	0.01	0.42	20883	3.47E-17
rs12694846	chr2:231148128	G	A	0.12	0.01	0.26	20883	2.50E-17
rs2270395	chr16:50846832	T	C	0.12	0.01	0.76	20883	8.93E-18
rs6556417	chr5:158823786	G	A	0.16	0.02	0.84	20883	2.05E-19
rs11117431	chr16:86015316	G	A	-0.15	0.02	0.20	20883	1.09E-19
rs17293632	chr15:67442596	T	C	0.13	0.01	0.24	20883	3.70E-20
rs438475	chr6:32186245	A	G	0.16	0.02	0.13	20883	3.42E-20
rs35164067	chr19:10525181	A	G	-0.14	0.02	0.20	20883	3.19E-20
rs516246	chr19:49206172	T	C	0.11	0.01	0.47	20883	1.33E-20
rs921720	chr8:126534671	G	A	0.12	0.01	0.62	20883	1.12E-21
rs13407913	chr2:25097644	G	A	0.11	0.01	0.43	20883	9.64E-22
rs780094	chr2:27741237	C	T	-0.12	0.01	0.61	20883	4.56E-22
rs6062496	chr20:62329099	A	G	0.12	0.01	0.57	20883	3.82E-22
rs13001325	chr2:102939036	T	C	-0.12	0.01	0.38	20883	1.68E-22
rs12411259	chr1:172866210	A	G	0.13	0.01	0.24	20883	1.43E-22
rs26528	chr16:28517709	C	T	0.12	0.01	0.46	20883	1.29E-22
rs7608910	chr2:61204856	G	A	0.12	0.01	0.39	20883	2.95E-23
rs6500315	chr16:50508101	G	A	0.15	0.01	0.78	20883	2.18E-23
rs9457247	chr6:167392174	T	C	0.12	0.01	0.54	20883	2.08E-23
rs8127691	chr21:45614860	C	T	-0.12	0.01	0.61	20883	4.48E-24
rs2024092	chr19:1124031	A	G	0.15	0.01	0.22	20883	7.13E-25
rs6561151	chr13:44484706	A	G	0.15	0.01	0.22	20883	4.68E-25
rs3024505	chr1:206939904	A	G	0.17	0.02	0.16	20883	3.95E-25
rs9889296	chr17:32570547	A	G	-0.14	0.01	0.27	20883	2.96E-25
rs11152949	chr6:106449085	G	A	0.13	0.01	0.32	20883	2.18E-25
rs1297258	chr21:16806709	T	C	-0.13	0.01	0.42	20883	2.11E-25

rs2847278	chr18:12778715	T	C	-0.17	0.02	0.84	20883	6.46E-26
rs1250573	chr10:81042475	A	G	-0.14	0.01	0.32	20883	5.86E-26
rs76906269	chr12:40607709	G	A	0.39	0.04	0.02	20883	1.75E-26
rs34779708	chr10:35466185	G	T	0.13	0.01	0.35	20883	1.90E-27
rs12131796	chr1:200878727	A	G	-0.15	0.01	0.28	20883	1.71E-27
rs7848647	chr9:117569046	C	T	0.14	0.01	0.67	20883	1.55E-27
rs4795397	chr17:38023745	G	A	0.13	0.01	0.47	20883	3.84E-28
rs9264942	chr6:31274380	C	T	0.15	0.01	0.35	20883	6.78E-32
rs3197999	chr3:49721532	A	G	0.16	0.01	0.28	20883	2.05E-33
rs10758669	chr9:4981602	A	C	-0.15	0.01	0.65	20883	4.19E-34
rs2413583	chr22:39659773	T	C	-0.21	0.02	0.17	20883	7.72E-36
rs11793497	chr9:139271850	G	A	0.17	0.01	0.42	20883	9.80E-44
rs7085798	chr10:101288347	A	C	-0.17	0.01	0.52	20883	1.53E-47
rs11236797	chr11:76299649	A	C	0.18	0.01	0.44	20883	8.54E-51
rs12796489[#]	chr11:3059360	A	C	-0.79	0.05	0.02	20883	4.96E-51
rs12766391	chr10:64441204	A	G	0.19	0.01	0.39	20883	9.59E-53
rs6111031	chr20:1682037	T	C	-0.28	0.02	0.16	20883	9.61E-55
rs17622378	chr5:131778452	G	A	0.19	0.01	0.42	20883	7.17E-56
rs7194886	chr16:50725193	T	C	-0.23	0.01	0.44	20883	1.42E-77
rs6738490	chr2:234161583	C	T	0.23	0.01	0.53	20883	4.26E-78
rs7711427*	chr5:40414886	C	A	0.25	0.01	0.61	20883	5.17E-88
rs7517847	chr1:67681669	G	T	-0.34	0.01	0.44	20883	1.38E-159

Note: *For rs7711427, not available from the outcome summary statistics of Parkinson's Disease; [#]for rs12796489, not available from the outcome summary statistics of Amyotrophic Lateral Sclerosis; chr:pos, position according to GRCh37/hg19 genome assembly.
Abbreviations: SNP, single nucleotide polymorphism; se, standard error; eaf, effect allele frequency.

Table S3. Characteristics of selected SNPs for ulcerative colitis (for validation).

SNP	chr:pos	effect_allele	other_allele	beta	se	eaf	samplesize	*P*-value
rs483905	chr11:96023427	A	G	0.05	0.02	0.29	26897	1.21E-08
rs6911490	chr6:106522027	C	T	-0.06	0.02	0.79	26897	1.46E-08
rs11739663	chr5:594083	C	T	-0.07	0.02	0.24	26897	1.81E-08
rs1728785	chr16:68591230	C	A	0.07	0.02	0.77	26897	3.71E-08
rs11597483	chr10:35285117	G	A	0.06	0.02	0.35	26897	3.68E-09
rs7184802	chr16:50355996	A	G	-0.09	0.02	0.18	26897	9.11E-09
rs7404095	chr16:23864590	C	T	0.07	0.02	0.57	26897	1.15E-08
rs4722672	chr7:27231762	T	C	-0.09	0.02	0.82	26897	2.06E-08
rs28374715	chr15:41563950	G	A	-0.08	0.02	0.26	26897	2.43E-08
rs2651244	chr1:70995562	A	G	-0.07	0.02	0.40	26897	2.29E-08
rs2472649	chr4:74857708	G	A	0.12	0.03	0.82	26897	2.57E-08
rs6437359	chr2:241597384	G	A	-0.08	0.02	0.74	26897	8.24E-09
rs4743820	chr9:93928416	T	C	0.08	0.02	0.70	26897	3.60E-09
rs6062504	chr20:62348907	G	A	0.08	0.02	0.68	26897	7.49E-11
rs11041476	chr11:1875067	A	G	0.08	0.02	0.32	26897	6.99E-10
rs12720356	chr19:10469975	C	A	0.13	0.03	0.09	26897	3.97E-10
rs1016883	chr2:198881668	A	G	-0.10	0.02	0.18	26897	2.87E-08
rs11612508	chr12:12657513	G	A	0.08	0.02	0.27	26897	1.06E-08

rs6088765	chr20:33799280	G	T	0.08	0.02	0.44	26897	2.21E-08
rs3742704	chr14:88477882	C	A	0.13	0.03	0.09	26897	1.55E-08
rs10797432	chr1:2501338	T	C	-0.08	0.02	0.48	26897	2.62E-12
rs11168249	chr12:48208368	C	T	0.08	0.02	0.47	26897	7.78E-09
rs6889364	chr5:40347469	A	G	0.12	0.03	0.11	26897	2.69E-11
rs1050152	chr5:131676320	T	C	0.08	0.02	0.43	26897	9.47E-11
rs2111485	chr2:163110536	G	A	-0.08	0.02	0.60	26897	1.93E-08
rs7523335	chr1:8180210	A	G	-0.11	0.02	0.18	26897	2.40E-13
rs7240004	chr18:46395022	G	A	-0.08	0.02	0.38	26897	1.31E-09
rs7210086	chr17:70641698	C	A	-0.11	0.02	0.20	26897	1.89E-09
rs941823	chr13:41013977	C	T	0.10	0.02	0.76	26897	2.95E-11
rs12103	chr1:1247494	C	T	-0.11	0.02	0.82	26897	2.73E-10
rs17539176	chr6:111777219	G	A	0.16	0.03	0.07	26897	1.54E-11
rs1026916	chr17:40529835	G	A	0.09	0.02	0.65	26897	2.79E-11
rs762422	chr21:45615638	A	G	-0.09	0.02	0.62	26897	4.76E-15
rs1512973	chr4:123506056	A	G	-0.09	0.02	0.33	26897	2.49E-10
rs11150589	chr16:30482494	C	T	-0.09	0.02	0.54	26897	6.04E-10
rs5763634	chr22:30350532	C	T	-0.10	0.02	0.74	26897	2.97E-11
rs11672983	chr19:55383051	A	G	0.09	0.02	0.39	26897	6.50E-11
rs2310173	chr2:102663628	G	T	-0.09	0.02	0.54	26897	3.89E-13
rs17694108	chr19:33731551	A	G	0.10	0.02	0.28	26897	8.16E-13
rs4728142	chr7:128573967	A	G	0.10	0.02	0.44	26897	4.37E-14
rs2155219	chr11:76299194	T	G	0.10	0.02	0.51	26897	1.79E-15
rs12933316	chr16:11699720	T	G	0.10	0.02	0.48	26897	8.52E-11
rs2413583	chr22:39659773	T	C	-0.15	0.02	0.17	26897	1.16E-13
rs561722	chr11:114386830	T	C	-0.11	0.02	0.34	26897	5.15E-17
rs3774959	chr4:103511114	A	G	0.11	0.02	0.36	26897	3.66E-12
rs1893217	chr18:12809340	G	A	0.14	0.02	0.16	26897	1.40E-12
rs798502	chr7:2789880	C	A	-0.12	0.02	0.29	26897	6.09E-17
rs17229285	chr2:199523122	T	C	-0.11	0.02	0.50	26897	1.73E-13
rs10761659	chr10:64445564	G	A	0.12	0.02	0.54	26897	4.95E-17
rs3749171	chr2:241569692	T	C	0.15	0.02	0.17	26897	3.07E-21
rs17085007	chr13:27531267	C	T	0.15	0.02	0.18	26897	2.79E-19
rs6920220	chr6:138006504	A	G	0.15	0.02	0.21	26897	1.40E-21
rs2823272[&]	chr21:16798586	A	T	-0.14	0.02	0.31	26897	5.90E-15
rs2816954[&]	chr1:200105746	A	T	0.21	0.03	0.85	26897	4.16E-15
rs4380874	chr7:107480315	C	T	-0.13	0.02	0.60	26897	2.06E-26
rs4263839	chr9:117566440	G	A	0.14	0.02	0.69	26897	5.44E-18
rs7608910	chr2:61204856	G	A	0.14	0.02	0.39	26897	6.57E-25
rs1422878	chr5:158839217	T	C	0.14	0.02	0.35	26897	5.40E-19
rs7134599	chr12:68500075	A	G	0.14	0.02	0.38	26897	8.51E-32
rs7554511	chr1:200877562	A	C	-0.16	0.02	0.28	26897	9.74E-25
rs10781499	chr9:139266405	A	G	0.15	0.02	0.41	26897	3.90E-28
rs10758669	chr9:4981602	A	C	-0.16	0.02	0.65	26897	6.96E-29
rs12946510	chr17:37912377	T	C	0.15	0.02	0.47	26897	3.39E-26
rs6584283	chr10:101290301	C	T	-0.16	0.02	0.52	26897	1.91E-34
rs3197999	chr3:49721532	A	G	0.19	0.02	0.30	26897	5.60E-36
rs1801274	chr1:161479745	G	A	-0.18	0.02	0.49	26897	2.12E-38

rs17396847	chr1:20144332	A	G	-0.18	0.02	0.39	26897	6.41E-26
rs3024505	chr1:206939904	A	G	0.24	0.02	0.16	26897	6.94E-41
rs6017342	chr20:43065028	C	A	0.21	0.02	0.53	26897	1.43E-43
rs2836878	chr21:40465534	A	G	-0.24	0.02	0.27	26897	4.62E-48
rs11209026	chr1:67705958	A	G	-0.52	0.04	0.07	26897	3.20E-62
rs6426833	chr1:20171860	A	G	0.24	0.02	0.54	26897	2.39E-68
rs6927022	chr6:32612397	G	A	-0.37	0.02	0.47	26897	4.71E-133

Note: [&]For rs2823272 and rs2816954, were excluded due to palindromic SNPs, when harmonizing the ulcerative colitis and Parkinson's Disease and Amyotrophic Lateral Sclerosis SNP effects; chr:pos, position according to GRCh37/hg19 genome assembly.

Abbreviations: SNP, single nucleotide polymorphism; se, standard error; eaf, effect allele frequency.

Table S4. Characteristics of selected SNPs for Crohn's disease (for validation).

SNP	chr:pos	effect_allele	other_allele	beta	se	eaf	samplesize	*P*-value
rs11264355[&]	chr1:155257492	G	C	0.06	0.02	0.25	30740	6.96E-09
rs8107703	chr19:10701259	A	G	-0.12	0.04	0.95	30740	4.01E-08
rs2790216	chr10:59997926	A	G	-0.07	0.02	0.22	30740	8.07E-09
rs7015630	chr8:90875918	C	T	-0.07	0.02	0.26	30740	1.42E-08
rs1363907	chr5:96252803	A	G	0.07	0.02	0.41	30740	5.61E-13
rs16967103	chr15:38899190	C	T	0.08	0.02	0.20	30740	3.88E-09
rs194749	chr14:69273905	C	T	0.08	0.02	0.23	30740	2.70E-10
rs630923	chr11:118754353	A	C	-0.09	0.02	0.15	30740	7.07E-09
rs10495903	chr2:43806918	T	C	0.11	0.02	0.13	30740	8.03E-12
rs10486483	chr7:26892440	A	G	0.09	0.02	0.25	30740	2.56E-08
rs2489623	chr6:127455821	C	A	0.07	0.02	0.52	30740	1.10E-09
rs1847472	chr6:90973159	A	C	-0.08	0.02	0.35	30740	1.57E-10
rs11177083	chr12:68571545	T	C	-0.08	0.02	0.47	30740	1.14E-08
rs6837335	chr4:48363983	G	A	0.08	0.02	0.65	30740	1.75E-08
rs17694108	chr19:33731551	A	G	0.09	0.02	0.28	30740	4.09E-09
rs7702331	chr5:72551134	G	A	-0.08	0.02	0.38	30740	5.63E-10
rs17391694	chr1:78623626	T	C	-0.13	0.03	0.11	30740	2.96E-09
rs714027	chr22:30577771	G	A	-0.08	0.02	0.54	30740	2.88E-12
rs17695092	chr5:173337853	G	T	-0.09	0.02	0.30	30740	4.68E-09
rs10127727	chr1:173150964	A	G	0.10	0.02	0.24	30740	6.51E-09
rs17293632	chr15:67442596	T	C	0.09	0.02	0.24	30740	5.97E-16
rs13126505	chr4:102865304	A	G	0.16	0.03	0.10	30740	2.33E-10
rs8014798	chr14:88456559	A	C	0.14	0.03	0.09	30740	1.53E-12
rs2029923	chr16:50916464	A	C	-0.08	0.02	0.59	30740	1.74E-10
rs12722515	chr10:6081230	A	C	-0.12	0.02	0.15	30740	3.76E-10
rs1042058	chr10:30728101	C	T	0.09	0.02	0.59	30740	5.93E-11
rs864745	chr7:28180556	C	T	-0.08	0.02	0.50	30740	3.65E-09
rs4145717	chr4:123316076	T	G	0.09	0.02	0.33	30740	3.14E-11
rs6142618	chr20:30725648	G	A	0.08	0.02	0.56	30740	2.30E-09
rs2227564	chr10:75673101	C	T	0.10	0.02	0.77	30740	6.75E-10
rs2162480	chr2:231130887	T	C	0.09	0.02	0.42	30740	6.67E-11
rs1517352	chr2:191931464	C	A	0.09	0.02	0.60	30740	1.93E-10
rs11227332	chr11:65623739	G	A	0.11	0.02	0.19	30740	2.62E-09
rs9466097	chr6:21462277	T	C	-0.09	0.02	0.47	30740	1.44E-11
rs10800309	chr1:161472158	G	A	-0.10	0.02	0.67	30740	6.38E-12

rs3897478	chr1:120451190	C	T	-0.15	0.03	0.11	30740	1.97E-11
rs6863411[&]	chr5:141513204	T	A	0.10	0.02	0.63	30740	3.59E-14
rs9557195	chr13:99956622	C	T	-0.11	0.02	0.23	30740	1.35E-12
rs1456896	chr7:50304461	T	C	0.10	0.02	0.69	30740	7.27E-15
rs1292053	chr17:57963537	G	A	0.09	0.02	0.45	30740	8.85E-13
rs259964	chr20:57824309	G	A	-0.09	0.02	0.54	30740	3.87E-11
rs2266959	chr22:21922904	T	G	0.12	0.02	0.19	30740	3.89E-16
rs10065637	chr5:55438851	T	C	-0.12	0.02	0.23	30740	3.68E-12
rs7725810	chr5:40827307	C	T	0.10	0.02	0.43	30740	1.46E-12
rs9313808	chr5:158820844	G	A	0.13	0.02	0.84	30740	4.88E-17
rs6879495	chr5:39979460	T	C	0.10	0.02	0.53	30740	4.53E-13
rs2488389	chr1:197631141	A	G	0.12	0.02	0.22	30740	8.45E-13
rs212388	chr6:159490436	T	C	-0.10	0.02	0.59	30740	3.04E-14
rs9358372	chr6:20812588	A	G	-0.10	0.02	0.62	30740	8.66E-14
rs6679677	chr1:114303808	A	C	-0.18	0.03	0.09	30740	2.02E-15
rs743479	chr21:45611950	T	C	-0.10	0.02	0.62	30740	1.76E-20
rs4246215	chr11:61564299	T	G	0.11	0.02	0.34	30740	1.92E-15
rs9286879	chr1:172862234	G	A	0.12	0.02	0.25	30740	5.52E-22
rs516246	chr19:49206172	T	C	0.10	0.02	0.48	30740	1.00E-15
rs4256159	chr3:18767404	T	C	0.14	0.02	0.14	30740	9.00E-15
rs921720	chr8:126534671	G	A	0.11	0.02	0.61	30740	8.30E-20
rs1870136	chr10:82307117	T	C	-0.15	0.02	0.30	30740	1.31E-10
rs12942547	chr17:40527544	G	A	-0.11	0.02	0.42	30740	3.34E-17
rs11879191	chr19:10512911	A	G	-0.13	0.02	0.20	30740	1.78E-17
rs6062504	chr20:62348907	G	A	0.12	0.02	0.68	30740	2.45E-19
rs13204742	chr6:128245765	T	G	0.16	0.02	0.12	30740	8.38E-15
rs1819333	chr6:167373547	G	T	-0.11	0.02	0.48	30740	6.76E-21
rs1734907	chr7:100315517	G	A	-0.15	0.02	0.85	30740	1.67E-13
rs1569723	chr20:44742064	A	C	-0.12	0.02	0.74	30740	9.95E-14
rs917997	chr2:103070568	C	T	-0.13	0.02	0.77	30740	3.12E-20
rs6651252	chr8:129567181	C	T	-0.17	0.03	0.14	30740	1.45E-16
rs3024505	chr1:206939904	A	G	0.15	0.02	0.16	30740	2.48E-19
rs2284553	chr21:34776695	G	A	0.12	0.02	0.60	30740	2.14E-16
rs1728918	chr2:27635463	G	A	-0.12	0.02	0.70	30740	4.86E-16
rs9265882	chr6:31313101	C	T	0.13	0.02	0.34	30740	5.58E-26
rs1250546	chr10:81032532	G	A	-0.12	0.02	0.40	30740	3.15E-18
rs6568421	chr6:106435025	G	A	0.13	0.02	0.30	30740	8.24E-20
rs7608910	chr2:61204856	G	A	0.12	0.02	0.39	30740	6.92E-19
rs4246905	chr9:117553249	C	T	0.14	0.02	0.71	30740	6.22E-26
rs2024092	chr19:1124031	A	G	0.14	0.02	0.22	30740	8.26E-22
rs529866	chr16:11373320	T	C	-0.16	0.02	0.20	30740	1.73E-16
rs11564258	chr12:40792300	A	G	0.38	0.05	0.03	30740	6.38E-29
rs6500315	chr16:50508101	G	A	0.15	0.02	0.77	30740	5.42E-23
rs3764147	chr13:44457925	G	A	0.14	0.02	0.25	30740	2.19E-21
rs26528	chr16:28517709	C	T	0.13	0.02	0.45	30740	9.65E-22
rs1893217	chr18:12809340	G	A	0.17	0.02	0.16	30740	3.05E-25
rs7554511	chr1:200877562	A	C	-0.14	0.02	0.28	30740	7.81E-19
rs2945412	chr17:25843643	A	G	0.13	0.02	0.59	30740	8.68E-17
rs6545800	chr2:25118885	T	C	0.13	0.02	0.45	30740	6.14E-16
rs3091316	chr17:32593974	A	G	-0.15	0.02	0.28	30740	1.22E-26

rs7532161	chr1:67642223	G	A	0.14	0.02	0.58	30740	1.90E-23
rs3197999	chr3:49721532	A	G	0.15	0.02	0.30	30740	5.31E-29
rs11010067[&]	chr10:35295431	G	C	0.15	0.02	0.35	30740	2.49E-25
rs907092	chr17:37922259	A	G	0.14	0.02	0.46	30740	2.69E-25
rs2823286	chr21:16817938	A	G	-0.17	0.02	0.29	30740	4.63E-28
rs10758669	chr9:4981602	A	C	-0.16	0.02	0.65	30740	1.44E-31
rs2413583	chr22:39659773	T	C	-0.22	0.02	0.17	30740	4.35E-31
rs11741861	chr5:150277909	G	A	0.28	0.03	0.09	30740	2.94E-37
rs4409764	chr10:101284237	G	T	-0.17	0.02	0.51	30740	1.80E-41
rs2155219	chr11:76299194	T	G	0.18	0.02	0.51	30740	9.56E-36
rs10761659	chr10:64445564	G	A	0.19	0.02	0.54	30740	6.37E-46
rs10781499	chr9:139266405	A	G	0.19	0.02	0.41	30740	3.48E-48
rs2188962	chr5:131770805	T	C	0.20	0.02	0.43	30740	1.35E-52
rs12994997	chr2:234173503	A	G	0.21	0.02	0.52	30740	4.14E-70
rs11742570	chr5:40410584	C	T	0.26	0.02	0.61	30740	1.81E-82
rs11209026	chr1:67705958	A	G	-0.86	0.04	0.07	30740	1.02E-146

Note: [&]For rs11264355, rs6863411 and rs11010067, were excluded due to palindromic SNPs, when harmonizing the Crohn's disease and Parkinson's Disease and Amyotrophic Lateral Sclerosis SNP effects; chr:pos, position according to GRCh37/hg19 genome assembly.
Abbreviations: SNP, single nucleotide polymorphism; se, standard error; eaf, effect allele frequency.

Table S5. Characteristics of selected SNPs for Parkinson's Disease

SNP	chr	pos	effect_allele	other_allele	beta	se	eaf	samplesize	*P*-value
rs35749011	1	155135036	A	G	0.75	0.07	0.02	482730	5.02E-30
rs823106	1	205656453	C	G	-0.15	0.02	0.85	482730	4.10E-10
rs4613239	2	169119609	G	C	0.18	0.02	0.13	482730	6.21E-13
rs6741007	2	135537119	G	T	-0.12	0.02	0.45	482730	2.09E-12
rs4488803	3	58218352	A	G	-0.11	0.02	0.37	482730	1.08E-08
rs10513789	3	182760073	G	T	-0.16	0.02	0.18	482730	3.18E-13
rs7695720	4	77183300	C	A	-0.13	0.02	0.21	482730	1.53E-09
rs34311866	4	951947	C	T	0.23	0.02	0.20	482730	7.97E-23
rs4698412	4	15737348	A	G	0.13	0.02	0.55	482730	7.05E-14
rs356203	4	90666041	T	C	-0.24	0.02	0.62	482730	3.01E-41
rs75646569	5	60345424	G	T	0.19	0.03	0.11	482730	5.62E-13
rs35265698[*]	6	32561334	G	C	-0.20	0.03	0.15	482730	3.93E-11
rs858295	7	23245569	G	A	-0.10	0.02	0.39	482730	3.83E-09
rs620490	8	16697579	G	T	-0.12	0.02	0.28	482730	6.46E-10
rs144814361	10	121410917	T	C	0.44	0.07	0.02	482730	9.07E-11
rs329647	11	133764666	C	G	-0.11	0.02	0.67	482730	1.94E-10
rs75505347	12	40885549	T	C	0.39	0.07	0.02	482730	6.12E-09
rs10847864	12	123326598	T	G	0.13	0.02	0.36	482730	9.81E-13
rs4774417	15	61993702	A	G	0.11	0.02	0.74	482730	4.63E-08
rs12934900	16	30923602	T	A	0.12	0.02	0.66	482730	4.33E-11
rs58879558	17	44095467	C	T	-0.24	0.03	0.22	482730	1.36E-21
rs10451230[#]	17	16035225	T	A	-0.10	0.02	0.57	482730	4.42E-08
rs4588066	18	40672964	A	G	0.10	0.02	0.33	482730	4.45E-09

Note: * For rs35265698, directly associate with ulcerative colitis ($P < 5\times10^{-8}$).
rs10451230 was palindromic with intermediate allele frequencies and were excluded as instrumental variables for ulcerative colitis and Crohn's disease.

Abbreviations: SNP, single nucleotide polymorphism; chr, chromosome; pos, position according to GRCh37/hg19 genome assembly; se, standard error; eaf, effect allele frequency.

Table S6. Characteristics of selected SNPs for Alzheimer's Disease

SNP	chr	pos	effect_allele	other_allele	beta	se	eaf	samplesize	*P*-value
rs4575098	chr1	161155392	A	G	0.02	0.00	0.23	455258	1.90E-10
rs679515	chr1	207750568	T	C	0.03	0.00	0.17	455258	6.83E-19
rs4663105	chr2	127891427	C	A	0.03	0.00	0.41	455258	1.45E-44
rs10933431	chr2	233981912	G	C	-0.02	0.00	0.24	455258	7.62E-10
rs6448453	chr4	11026028	A	G	0.01	0.00	0.26	455258	1.98E-09
rs6931277*	chr6	32583357	T	A	-0.02	0.00	0.16	455258	7.35E-11
rs9381563	chr6	47432637	C	T	0.01	0.00	0.36	455258	1.99E-10
rs1859788	chr7	99971834	A	G	-0.02	0.00	0.32	455258	1.80E-15
rs7810606	chr7	143108158	T	C	-0.01	0.00	0.49	455258	2.89E-11
rs755951	chr8	27226790	C	A	0.02	0.00	0.41	455258	1.13E-11
rs4236673	chr8	27464929	A	G	-0.02	0.00	0.38	455258	1.48E-19
rs11257238	chr10	11717397	C	T	0.01	0.00	0.36	455258	1.04E-08
rs2081545	chr11	59958380	A	C	-0.02	0.00	0.38	455258	1.11E-15
rs867611	chr11	85776544	G	A	-0.02	0.00	0.32	455258	1.48E-18
rs11218343	chr11	121435587	C	T	-0.04	0.01	0.04	455258	8.12E-12
rs12590654	chr14	92938855	A	G	-0.01	0.00	0.34	455258	1.32E-10
rs442495	chr15	59022615	C	T	-0.01	0.00	0.35	455258	1.22E-09
rs59735493	chr16	31133100	A	G	-0.01	0.00	0.30	455258	3.73E-08
rs113260531	chr17	5138980	A	G	0.02	0.00	0.13	455258	7.91E-10
rs28394864	chr17	47450775	A	G	0.01	0.00	0.45	455258	1.68E-08
rs111278892	chr19	1039323	G	C	0.02	0.00	0.15	455258	6.67E-11
rs4516335	chr19	45217655	C	T	-0.02	0.00	0.11	455258	5.16E-09
rs12972156	chr19	45387459	G	C	0.17	0.00	0.13	455258	0
rs117310449	chr19	45393516	T	C	0.22	0.01	0.01	455258	2.15E-94
rs117316645	chr19	45458212	A	G	0.06	0.01	0.04	455258	4.78E-24
rs204473	chr19	45487519	A	G	-0.04	0.01	0.02	455258	2.58E-09
rs6014724	chr20	54998544	G	A	-0.02	0.00	0.10	455258	5.38E-10

Note: * For rs6931277, directly associate with ulcerative colitis ($P < 5\times10^{-8}$).

Abbreviations: SNP, single nucleotide polymorphism; chr, chromosome; pos, position according to GRCh37/hg19 genome assembly; se, standard error; eaf, effect allele frequency.

Table S7. Characteristics of selected SNPs for Amyotrophic Lateral Sclerosis.

SNP	chr	pos	effect_allele	other_allele	beta	se	eaf	samplesize	*P*-value
rs10463311	5	150410835	T	C	-0.09	0.02	0.74	80610	4.00E-08
rs3849943	9	27543382	T	C	-0.18	0.02	0.75	80610	3.77E-30
rs142321490	12	58676132	C	G	0.32	0.05	0.02	80610	6.15E-10
rs74654358	12	64881967	A	G	0.20	0.03	0.05	80610	4.66E-09
rs12973192	19	17753239	G	C	0.12	0.02	0.32	80610	3.92E-15
rs75087725	21	45753117	A	C	0.51	0.07	0.02	80610	1.85E-14

Abbreviations: SNP, single nucleotide polymorphism; chr, chromosome; pos, position according to GRCh37/hg19 genome assembly; se, standard error; eaf, effect allele frequency.

Table S8. F-statistics for the exposure variables.

**Mendelian randomization analysis**	**Trait**	R^2 （%）	***F*-statistic**
primary	ulcerative colitis	12.14	44.47
	Crohn's disease	24.62	59.50
validation	ulcerative colitis	6.07	23.76
	Crohn's disease	8.19	27.06
reverse causality	Parkinson's Disease	0.13	35.92
	Alzheimer's disease	0.48	82.11
	Amyotrophic Lateral Sclerosis	0.22	29.44

Abbreviations: Abbreviations: R^2, variance of exposure explained by selected instrumental variables.

Table S9. Associations between genetically predicted inflammatory bowel disease (ulcerative colitis and Crohn's disease) and neurodegenerative diseases in Mendelian randomization analyses.

IBD	NDs	MR method	Number of SNPs	*OR (95% CI)*	*P*-value	Cochran *Q* statistic	Heterogeneity *P*-value	MR-Egger intercept	*P*-value
UC	PD	Inverse variance weighted	84	1.01 (0.96,1.06)	0.65	174.96	1.59E-08		
		MR Egger	84	0.99 (0.87,1.12)	0.85	174.59	1.19E-08	3.40E-03	0.68
		Weighted median	84	1.01 (0.95,1.07)	0.81				
		Simple mode	84	1.02 (0.90,1.14)	0.77				
		Weighted mode	84	1.04 (0.96,1.12)	0.36				
UC[a]	PD	Inverse variance weighted	81	1.01 (0.97,1.05)	0.63	112.65	9.48E-03		
		MR Egger	81	1.02 (0.93,1.13)	0.64	112.52	7.90E-03	-2.00E-03	0.77
		Weighted median	81	1.01 (0.96,1.07)	0.71				
		Simple mode	81	1.02 (0.91,1.14)	0.77				
		Weighted mode	81	1.03 (0.96,1.11)	0.38				
CD	PD	Inverse variance weighted	113	1.01 (0.97,1.05)	0.54	214.33	2.06E-08		
		MR Egger	113	1.08 (0.97,1.21)	0.17	211.36	3.05E-08	-9.43E-03	0.21
		Weighted median	113	1.01 (0.96,1.07)	0.70				
		Simple mode	113	1.05 (0.93,1.19)	0.45				
		Weighted mode	113	1.01 (0.94,1.10)	0.72				
CD[b]	PD	Inverse variance weighted	108	1.00 (0.97,1.04)	0.95	143.95	9.99E-03		
		MR Egger	108	1.02 (0.93,1.13)	0.63	143.62	8.81E-03	-3.21E-03	0.62
		Weighted median	108	1.01 (0.96,1.06)	0.75				
		Simple mode	108	1.05 (0.93,1.18)	0.43				
		Weighted mode	108	1.01 (0.94,1.09)	0.76				
UC	AD	Inverse variance weighted	85	1.00 (0.99,1.00)	0.57	120.80	5.29E-03		
		MR Egger	85	1.01 (1.00,1.01)	0.25	117.39	7.76E-03	-1.21E-03	0.12
		Weighted median	85	1.00 (0.99,1.00)	0.31				
		Simple mode	85	1.01 (0.99,1.01)	0.43				
		Weighted mode	85	0.99 (0.98,0.99)	0.22				
CD	AD	Inverse variance weighted	114	1.00 (0.99,1.00)	0.26	164.54	1.12E-03		

		MR Egger	114	1.00 (0.98,1.00)	0.49	164.41	9.29E-04	2.32E-04	0.77
		Weighted median	114	1.00 (0.99,1.00)	0.19				
		Simple mode	114	0.99 (0.98,0.99)	0.17				
		Weighted mode	114	1.00 (0.99,1.00)	0.31				
UC	ALS	Inverse variance weighted	84	0.98 (0.96,1.01)	0.24	97.06	1.39E-01		
		MR Egger	84	1.00 (0.93,1.07)	0.97	96.68	1.28E-01	-2.58E-03	0.57
		Weighted median	84	0.99 (0.94,1.03)	0.49				
		Simple mode	84	0.98 (0.89,1.07)	0.61				
		Weighted mode	84	0.97 (0.90,1.04)	0.40				
CD	ALS	Inverse variance weighted	113	0.99 (0.96,1.02)	0.41	159.82	2.04E-03		
		MR Egger	113	1.02 (0.95,1.10)	0.53	158.40	2.12E-03	-4.90E-03	0.32
		Weighted median	113	0.99 (0.96,1.03)	0.61				
		Simple mode	113	1.03 (0.94,1.12)	0.53				
		Weighted mode	113	1.03 (0.97,1.08)	0.38				

Note: [a] Remove the outliers (rs1801274, rs76904798, rs1297256) identified by MR-PRESSO. [b] remove the outliers (rs11175963, rs7194886, rs780094, rs1297258, rs727563) that identified by MR-PRESSO.

Abbreviations: IBD, Inflammatory bowel disease ; NDs, Neurodegenerative diseases; MR, Mendelian randomization; UC, ulcerative colitis; CD, Crohn's disease; AD, Alzheimer's disease; PD, Parkinson's Disease; ALS, Amyotrophic Lateral Sclerosis.

Table S10. Associations between genetically predicted inflammatory bowel disease (ulcerative colitis and Crohn's disease, for validation) and neurodegenerative diseases in Mendelian randomization analyses.

IBD	NDs	MR method	Number of SNPs	*OR (95% CI)*	*P*-value	Cochran *Q* statistic	Heterogeneity p-value	MR-Egger intercept	*P*-value
UC	PD	Inverse variance weighted	71	0.98 (0.94,1.02)	0.24	124.51	3.14E-02		
		MR Egger	71	0.97 (0.89,1.07)	0.60	123.77	2.97E-02	2.91E-04	0.96
		Weighted median	71	1.00 (0.95,1.06)	0.88				
		Simple mode	71	1.02 (0.92,1.13)	0.72				
		Weighted mode	71	1.00 (0.94,1.07)	0.89				
CD	PD	Inverse variance weighted	98	0.99 (0.96,1.03)	0.75	124.51	3.14E-02		

		MR Egger	98	1.02 (0.95,1.10)	0.59	123.77	2.97E-02	-4.02E-03	0.45
		Weighted median	98	1.01 (0.96,1.06)	0.79				
		Simple mode	98	1.00 (0.89,1.12)	0.98				
		Weighted mode	98	1.00 (0.95,1.06)	0.88				
			98						
UC	AD	Inverse variance weighted	73	1.00 (0.99,1.00)	0.29	109.77	2.76E-03		
		MR Egger	73	1.01 (0.99,1.01)	0.35	106.39	4.17E-03	-1.33E-03	0.14
		Weighted median	73	1.00 (0.99,1.00)	0.25				
		Simple mode	73	1.00 (0.98,1.00)	0.83				
		Weighted mode	73	1.00 (0.99,1.00)	0.46				
CD	AD	Inverse variance weighted	101	1.00 (0.99,1.00)	0.06	155.74	3.05E-04		
		MR Egger	101	1.00 (0.99,1.00)	0.71	155.43	2.54E-04	-3.30E-04	0.66
		Weighted median	101	1.00 (0.99,1.00)	0.30				
		Simple mode	101	0.99 (0.98,0.99)	0.23				
		Weighted mode	101	0.99 (0.99,0.99)	0.16				
UC	ALS	Inverse variance weighted	71	0.98 (0.95,1.01)	0.17	62.74	7.19E-01		
		MR Egger	71	1.00 (0.93,1.06)	0.90	62.49	6.97E-01	-2.18E-03	0.62
		Weighted median	71	0.98 (0.94,1.02)	0.37				
		Simple mode	71	0.95 (0.86,1.04)	0.24				
		Weighted mode	71	0.95 (0.89,1.02)	0.14				
CD	ALS	Inverse variance weighted	98	0.98 (0.96,1.01)	0.23	127.43	2.08E-02		
		MR Egger	98	0.98 (0.92,1.04)	0.42	127.29	1.80E-02	1.40E-03	0.74
		Weighted median	98	0.98 (0.94,1.02)	0.29				
		Simple mode	98	0.99 (0.92,1.06)	0.75				
		Weighted mode	98	0.98 (0.93,1.02)	0.34				

Abbreviations: IBD, Inflammatory bowel disease ; NDs, Neurodegenerative diseases; MR, Mendelian randomization; UC, ulcerative colitis; CD, Crohn's disease; AD, Alzheimer's disease; PD, Parkinson's Disease; ALS, Amyotrophic Lateral Sclerosis.

Table S11. Associations between genetically predicted neurodegenerative diseases and ulcerative colitis and Crohn's disease in Mendelian randomization analyses.

Exposures	Outcomes	MR method	Number of SNPs	*OR* (95% *CI*)	*P*-value
Parkinson's Disease	ulcerative colitis	Inverse variance weighted	21	1.01 (0.94,1.08)	0.84
		MR Egger	21	0.94 (0.77,1.13)	0.50
		Weighted median	21	1.05 (0.95,1.17)	0.37
		Simple mode	21	1.06 (0.88,1.27)	0.53
		Weighted mode	21	1.08 (0.94,1.24)	0.29
Parkinson's Disease	Crohn's disease	Inverse variance weighted	22	1.08 (0.96,1.21)	0.21
		MR Egger	22	1.12 (0.82,1.53)	0.49
		Weighted median	22	0.98 (0.87,1.10)	0.73
		Simple mode	22	0.97 (0.77,1.21)	0.77
		Weighted mode	22	0.93 (0.79,1.10)	0.41
Alzheimer's disease	ulcerative colitis	Inverse variance weighted	26	1.14 (0.81,1.60)	0.44
		MR Egger	26	1.24 (0.79,1.97)	0.36
		Weighted median	26	1.27 (0.86,1.87)	0.24
		Simple mode	26	2.00 (0.66,6.11)	0.23
		Weighted mode	26	1.28 (0.90,1.82)	0.18
Alzheimer's disease	Crohn's disease	Inverse variance weighted	27	1.20 (0.80,1.82)	0.38
		MR Egger	27	1.08 (0.62,1.90)	0.78
		Weighted median	27	1.14 (0.76,1.72)	0.52
		Simple mode	27	0.82 (0.30,2.29)	0.71
		Weighted mode	27	1.15 (0.75,1.75)	0.52
Amyotrophic Lateral Sclerosis	ulcerative colitis	Inverse variance weighted	6	1.03 (0.87,1.22)	0.72
		MR Egger	6	1.02 (0.67,1.54)	0.94
		Weighted median	6	1.07 (0.87,1.31)	0.51
		Simple mode	6	1.12 (0.83,1.51)	0.51
		Weighted mode	6	1.11 (0.85,1.44)	0.49
Amyotrophic Lateral Sclerosis	Crohn's disease	Inverse variance weighted	6	0.95 (0.79,1.14)	0.59
		MR Egger	6	1.13 (0.72,1.76)	0.63

	Weighted median	6	0.97 (0.78,1.20）	0.77
	Simple mode	6	0.94 (0.66,1.35）	0.76
	Weighted mode	6	1.03 (0.77,1.37）	0.87

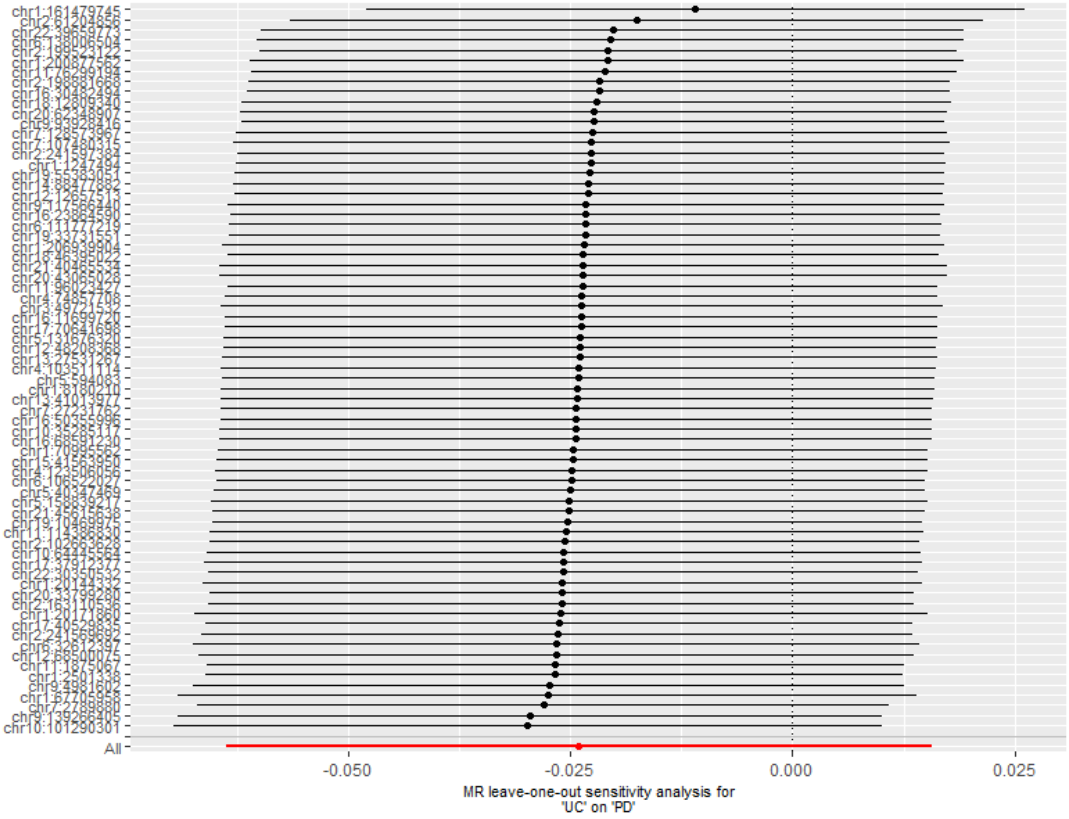

Figure S1. Leave-one-out plot presenting the relationship between ulcerative colitis and Parkinson's Disease

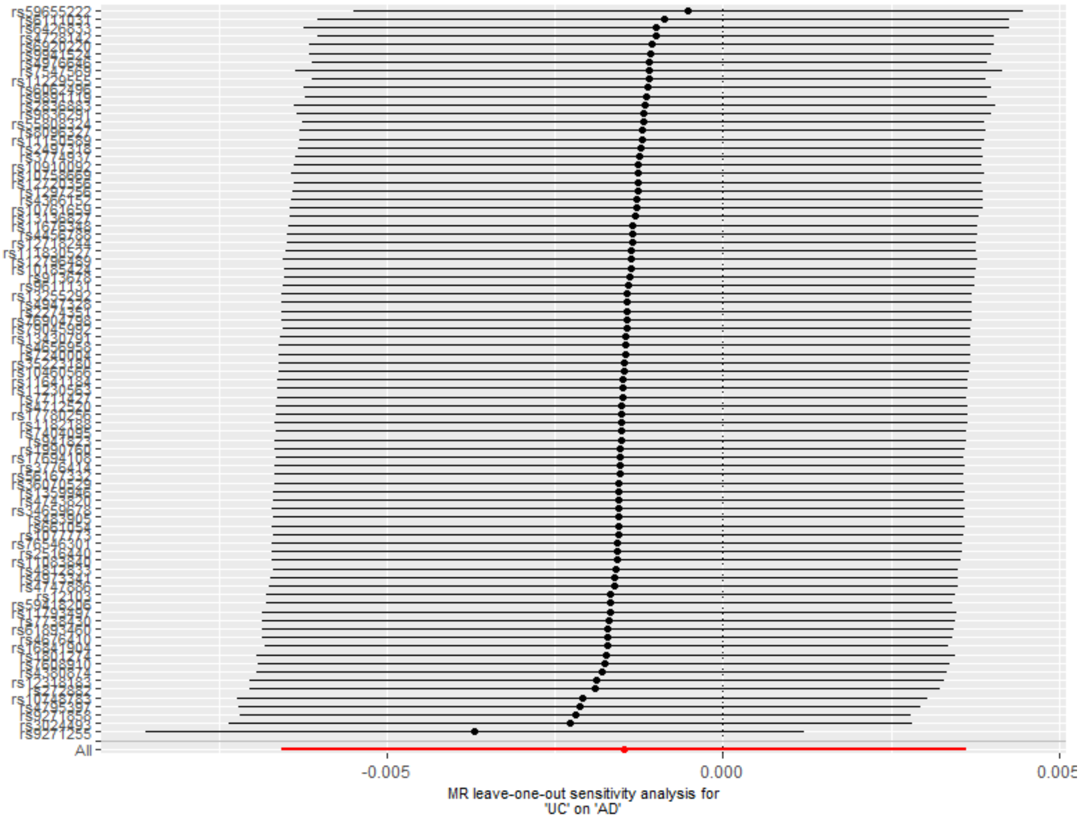

Figure S2. Leave-one-out plot presenting the relationship between ulcerative colitis and Alzheimer's Disease.

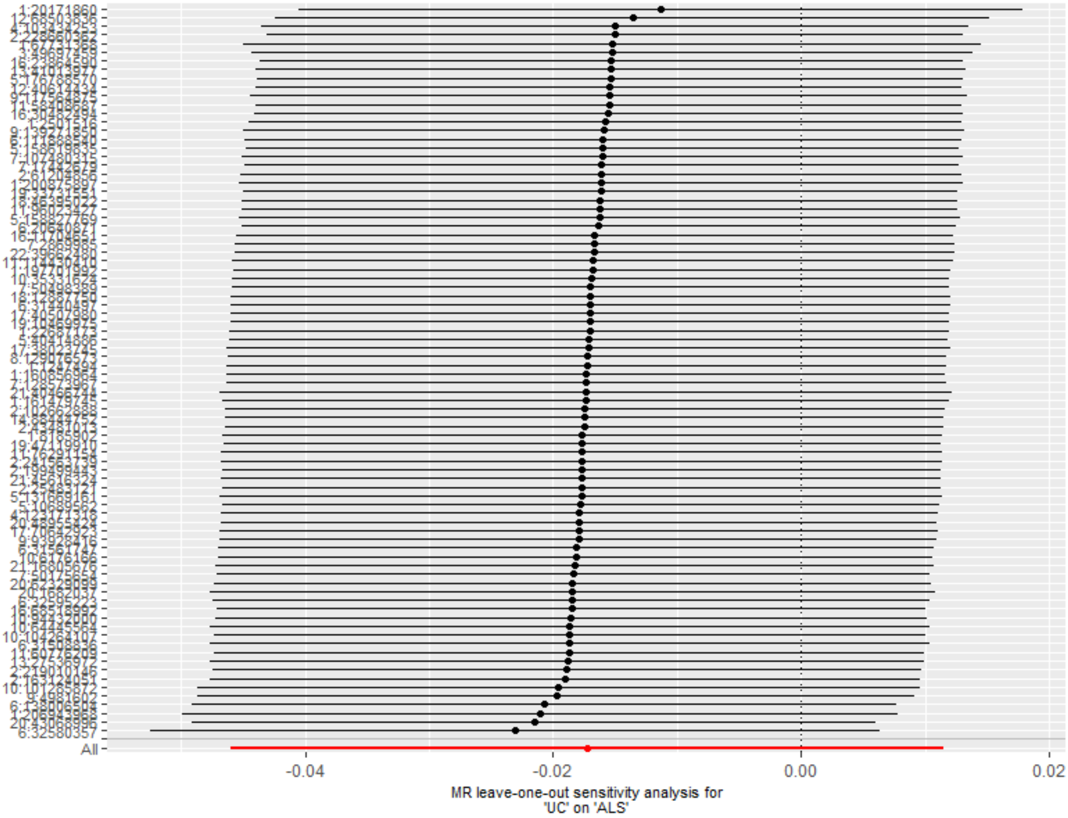

Figure S3. Leave-one-out plot presenting the relationship between ulcerative colitis and Amyotrophic Lateral Sclerosis.

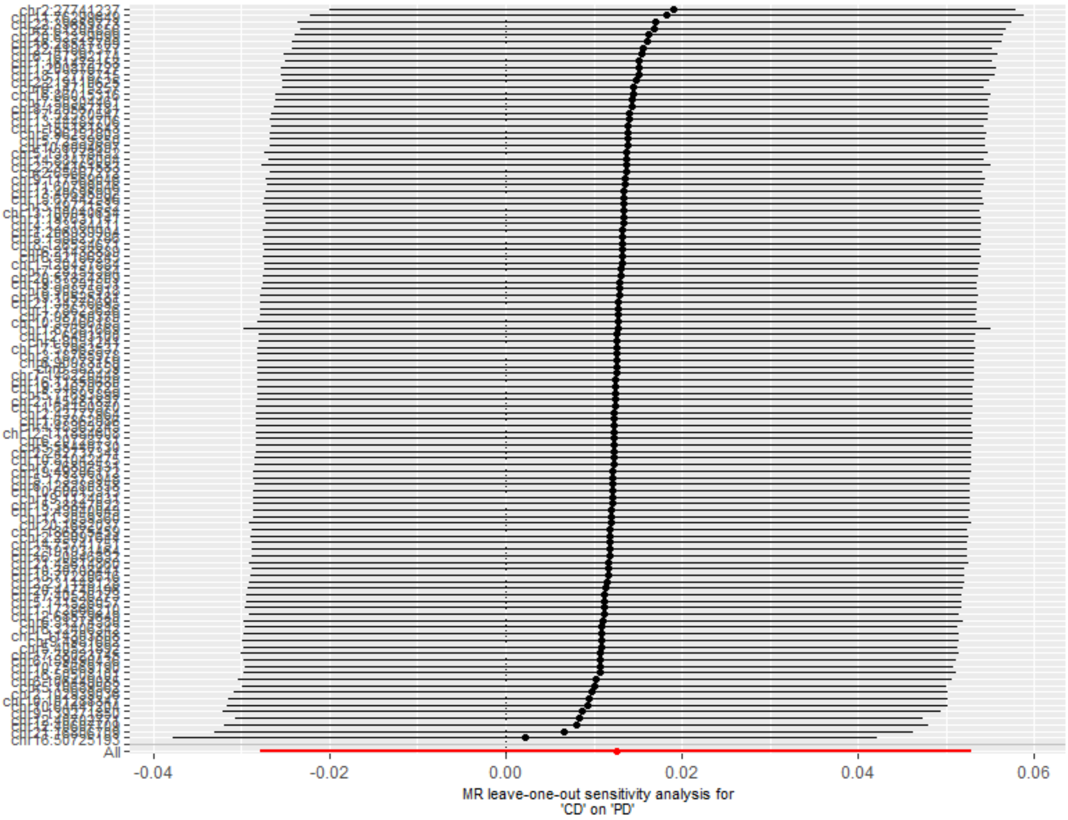

Figure S4. Leave-one-out plot presenting the relationship between Crohn's disease and Parkinson's Disease

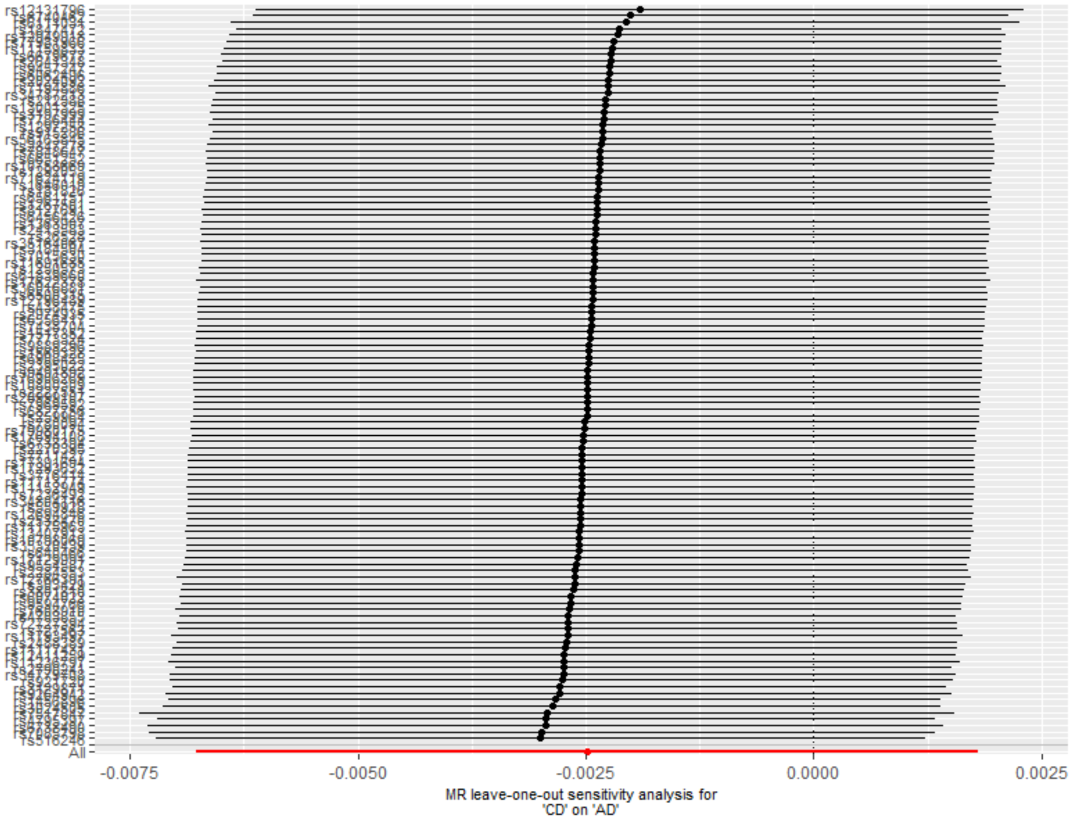

Figure S5. Leave-one-out plot presenting the relationship between Crohn's disease and Alzheimer's Disease.

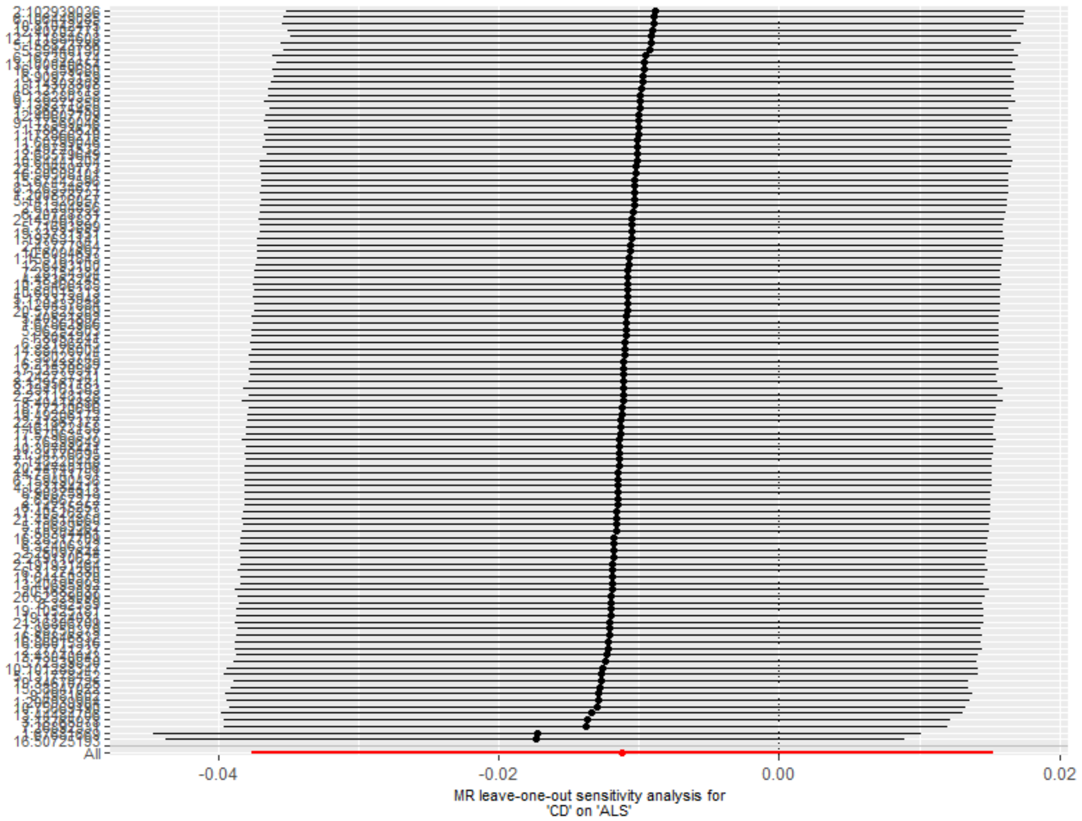

Figure S6. Leave-one-out plot presenting the relationship between Crohn's disease and Amyotrophic Lateral Sclerosis.
